# Associations of intakes of total protein, protein from dairy sources, and dietary calcium with risks of colorectal, breast, and prostate cancer: a prospective analysis in UK Biobank

**DOI:** 10.1038/s41416-023-02339-2

**Published:** 2023-07-05

**Authors:** Cody Z. Watling, Rebecca K. Kelly, Yashvee Dunneram, Anika Knuppel, Carmen Piernas, Julie A. Schmidt, Ruth C. Travis, Timothy J. Key, Aurora Perez-Cornago

**Affiliations:** 1 Cancer Epidemiology Unit, Nuffield Department of Population Health, University of Oxford, Oxford, United Kingdom; 2 MRC Unit of Lifelong Health and Ageing, University College London, London, United Kingdom; 3 Nuffield Department of Primary Care, University of Oxford, Oxford, United Kingdom; 4 Department of Clinical Epidemiology, Department of Clinical Medicine, Aarhus University and Aarhus University Hospital, Aarhus, Denmark

**Keywords:** Risk factors, Oncology

## Abstract

**Background:**

Evidence concerning intakes of protein or sources of dairy protein and risks of colorectal, breast, and prostate cancers is inconclusive.

**Methods:**

Using a subsample of UK Biobank participants who completed ≥2 (maximum of 5) 24-h dietary assessments, we estimated intakes of total protein, protein from total dairy products, milk, and cheese, and dietary calcium in 114,217 participants. Hazard ratios (HRs) and 95% confidence intervals (CI) were estimated using multivariable-adjusted Cox regression.

**Results:**

After a median of 9.4 years of follow-up, 1193 colorectal, 2024 female breast, and 2422 prostate cancer cases were identified. There were inverse associations of total dairy protein, protein from milk, and dietary calcium intakes with colorectal cancer incidence (HR_Q4 vs Q1_:0.80, 95% CI: 0.67–0.94; 0.79, 0.67–0.94; 0.71, 0.58–0.86, respectively). We also observed positive associations of milk protein and dietary calcium with prostate cancer risk (HR_Q4 vs Q1_:1.12, 1.00–1.26 and 1.16, 1.01–1.33, respectively). No significant associations were observed between intake of dairy protein and breast cancer risk. When insulin-like growth factor-I concentrations measured at recruitment were added to the multivariable-adjusted models, associations remained largely unchanged. Analyses were also similar when looking at total grams of dairy products, milk, and cheese.

**Conclusion:**

Further research is needed to understand the mechanisms underlying the relationships of dairy products with cancer risk and the potential roles of dietary protein and calcium.

## Introduction

Colorectal, breast, and prostate cancer are among the most commonly diagnosed cancers in the world, accounting for approximately 28% of all new cancer cases in 2020 [1]. The most recent World Cancer Research Fund (WCRF) meta-analysis for colorectal cancer, which included studies published up until 2017, reported that a higher intake of total dairy products is associated with a “probable” lower risk of colorectal cancer [2, 3], possibly due to the high calcium content in dairy products. The findings from the 2017 WCRF meta-analysis on breast cancer classified the evidence for an inverse association between the intake of dairy products and risk of breast cancer as “limited-suggestive” for premenopausal breast cancer but “limited-no conclusion” for postmenopausal breast cancer [4, 5]. The latest WCRF meta-analysis for prostate cancer, which included studies up until 2014, reported “limited-suggestive” evidence that higher intake of dairy products may be associated with a higher risk of overall prostate cancer [6, 7]. However, the WCRF meta-analyses did not look at protein from dairy products or include separate analyses for different dairy protein sources, and few studies to date have assessed the relationship of protein from dairy products with these cancer sites [8–13]. This may be important because there is some evidence that protein, particularly from dairy products, increases circulating concentrations of insulin-like-growth factor-I (IGF-I) [14, 15], a peptide hormone which stimulates cell growth and proliferation [16].

Both observational and Mendelian randomisation (MR) studies have found that the risks of developing colorectal, breast, and prostate cancer are associated with higher circulating concentrations of IGF-I [17–21]. In cross-sectional studies and in some randomised controlled trials (RCTs), protein intake, particularly from dairy products, has been positively associated with circulating IGF-I concentrations [14, 22, 23]. Most studies [15, 24, 25] (but not all [26]) that have investigated associations between dairy product source and IGF-I concentrations suggest that the association might be restricted to protein from milk but not from cheese; however, it remains unclear if this possible differing association of dairy product intake with IGF-I may be important for the aetiology of IGF-I related cancers.

Therefore, this study aimed to assess the associations of intakes of total protein, dairy protein, sources of dairy protein, and total dietary calcium with risks of colorectal, breast, and prostate cancer in a large British cohort. A secondary aim was to assess the potential role of IGF-I concentrations in these associations.

## Methods

### Study design and participants

In total, over 500,000 individuals (5.5% response rate) aged 37–73 years consented to take part in the UK Biobank study from 2006 to 2010 [27]. In brief, using the National Health Service patient registries a total of 9.2 million people, living within 40 km of an assessment centre, were invited to participate by attending a visit at one of the 22 assessment centres around the United Kingdom (UK). At recruitment, participants provided informed consent and provided data via a self-administered touchscreen questionnaire and computer assisted personal interview regarding their lifestyle, sociodemographic information, and reproductive history. Anthropometric measurements were also taken using standardised procedures by a trained professional [28], and blood samples were collected from participants [29]. Information on covariate data collection and classification can be found in the Supplementary Material.

Ethical approval for the UK Biobank study was obtained from the North West Multi-Centre Research Ethics Committee (reference number 21/NW/0157). A full description of the study recruitment, assessment, protocol, and ethical approval can be found on the UK Biobank website [30].

### Assessment of diet: 24-h dietary assessment

Dietary intake was assessed in a subsample of participants using a validated web-based 24-h dietary assessment, the Oxford WebQ [31, 32]. This dietary assessment asked participants to recall the frequency of consumption of 206 types of foods and 32 types of drinks during the previous 24 h [33]. The Oxford WebQ was shown to capture dietary intakes similarly to 24-h interviewer recalls (mean Spearman correlation between the two assessments for 21 nutrients was 0.6 with the majority of nutrients having a correlation between 0.5 and 0.9) [33] and was validated in a sample of 160 individuals using biomarkers; total protein intake estimated by two 24-h dietary assessments was determined to have a correlation of 0.47 (95% confidence interval (CI): 0.33–0.61) with a recovery biomarker for protein intake estimated from urinary nitrogen excretion [34], with higher correlations when more 24-h dietary assessments were completed.

The Oxford WebQ 24-h dietary assessment was completed at recruitment by the last 70,474 participants recruited to the UK Biobank study, between April 2009 and September 2010. For all participants who provided a valid email address at recruitment (*n* = 331,013 of the total UK Biobank sample), the 24-h dietary assessment was also sent every 3–4 months for a total of four times between February 2011 and June 2012 (First cycle: February 2011 to April 2011; Second cycle: June 2011 to September 2011; Third cycle: October 2011 to December 2011; Fourth cycle: April 2012 to June 2012; Supplementary Fig. 1). Invitation emails were sent on variable days of the week and participants in the first or second cycle were given 3 days to complete the 24-h dietary assessment whereas for cycles three and four, 14 days were provided to click the link to complete the dietary assessment before it expired. The response rate for the follow-up 24-h dietary assessments ranged from 26 to 33%.

### Estimation of nutrient intake

Total protein, dairy protein, and total dietary calcium intakes were estimated for each 24-h dietary assessment based on food and beverage intakes reported by participants. From these responses, intakes of total protein, protein from all dairy products (milk, yogurt, cheese, cream, butter, dairy desserts, and dairy drinks combined), milk, and cheese, as well as total dietary calcium, were calculated by multiplying the protein or calcium content of each respective food and beverage by the frequency of intake using the UK Nutrient Databank food composition tables [31, 32]. Percentages of energy from total protein, protein from all dairy sources, and separately from milk and cheese intake, as well as total mg of calcium intake, were estimated for each 24-h dietary assessment and then averaged across all available 24-h dietary assessments. It was not possible to assess protein intake from other individual dairy sources, such as yogurt, due to the large number of participants that did not consume these products on the day(s) of dietary assessment. Full description of the calculation of intake of total protein, protein from all dairy products and dairy sources, and total dietary calcium can be found in the Supplementary Methods. We also report results for estimated absolute grams per day (g/day) of all dairy products, milk, and cheese reported from all 24-h dietary assessments for comparison with previous meta-analyses [2, 4, 6].

### Outcome ascertainment: cancer diagnoses

Prevalent and incident cancer diagnoses were determined from a combination of linkage to National Health Service Digital (for participants from England and Wales), Hospital Episode Statistics (HES) data for English participants, and National Health Service Central Register Scotland and Scottish Morbidity Records (SMR) for Scottish participants [35]. Participants contributed follow-up time from date of completion of their last 24-h dietary assessment until the date of first cancer registration (excluding non-melanoma skin cancer (International Statistical Classification of Disease (ICD-10) code: C44)), date of death, or last date of follow-up available from HES and SMR data or the Welsh cancer registry (30^th^ of September 2021 for English participants, 31^st^ of July 2021 for Scottish participants, and 29^th^ of February 2020 for Welsh participants). Cancer registry data were available until 29^th^ of February 2020 for English participants and 31^st^ of January 2021 for Scottish participants, after this time participants from England and Scotland were followed using HES and SMR databases, respectively. For participants from Wales, hospital episode data were not used because the cancer registry had longer follow-up. Participants were coded as having a diagnosis of cancer based on the ICD-10 codes of their first incident cancer: colorectal cancer (C18-C20), female breast cancer (C50), or prostate cancer (C61).

### Exclusions

A total of 922 participants had withdrawn their consent from the UK Biobank and were excluded from this analysis. Participants were also excluded if they had been diagnosed with a prevalent malignant cancer before recruitment (excluding non-melanoma skin cancer (*N* = 29,504)), their genetic sex did not match their reported sex (*N* = 321), or they did not complete a 24-h dietary assessment (*N* = 251,938). Individual 24-h dietary assessments were excluded if an unreliable energy intake was reported (men: >17,575 kJ (4200 kcal) or <3347 kJ (800 kcal); women >14,644 kJ (3500 kcal) or <2092 kJ (500 kcal)) [36] or the participant reported that they were ill or fasting on the day they completed the questionnaire (*N* = 2439 reported unreliable energy intake and no longer had a 24-h dietary assessment, *N* = 592 were ill or fasting and no longer had a valid 24-h dietary assessment). To reduce random measurement error, participants who did not complete a minimum of two valid 24-h dietary assessments were excluded (*N* = 100,487). Participants were also excluded if they were censored or had a cancer diagnosis before the completion of their last 24-h dietary assessment (*N* = 2897). In total, this left 114,217 participants who completed ≥2 (maximum 5) valid 24-h dietary assessments, of which 51,278 were men and 62,939 were women. Supplementary Fig. 2 shows the flow diagram of exclusions for these analyses. Of the 114,217 included participants, 44,994 completed two 24-h dietary assessments, 38,480 completed three, 26,032 completed four and 4711 completed five (Supplementary Fig. 1).

### Statistical analyses

Participants were classified into quartiles of percentage of energy intake from total protein, protein from all dairy products, protein from milk, protein from cheese, except for total dietary calcium intake which was expressed in quartiles of mg/day. Intakes of total protein and protein from all dairy products and sources of dairy protein were also modelled as a per 2.5% energy increment whereas total dietary calcium intake was modelled as a 300 mg/day increment. Baseline characteristics were summarised across quartiles of percentage of energy intake from total protein, protein from all dairy, protein from milk, protein from cheese, as well as total dietary calcium intake.

Cox proportional hazards regressions were used to estimate hazard ratios (HRs) and 95% CIs, with age as the underlying time variable. When breast cancer was the outcome of interest, models were restricted to women, and when prostate cancer was the outcome, models were restricted to men. The lowest quartile of percentage of energy intake of total protein, dairy protein, or dietary calcium intake (in mg/day) was used as the reference group for analyses by quartile of intake. Minimally adjusted models were stratified by age at recruitment (<45, 45–49, 50–54, 55–59, 60–64, ≥65 years) and by sex (only for analyses with colorectal cancer as the outcome), and adjusted for region at recruitment (North-West England, North-Eastern England, Yorkshire & the Humber, West Midlands, East Midlands, South-East England, South-West England, London, Wales, and Scotland).

Multivariable Cox regression analyses were further adjusted for height (six sex-specific categories increasing by 5 cm, or unknown/missing (0.16%)), physical activity (low: 0–9.99, medium: 10–49.99, high: ≥50 excess metabolic equivalent of task-hours/week, or unknown/missing (1.81%)), Townsend deprivation index (quintiles from most deprived to least deprived, and unknown/missing (0.12%)), education (completion of national exam at 16 years, completion of national exam at 17–18 years, college or university degree, or other/unknown/missing (6.6%)), employment status (employed, retired, not in paid employment, or unknown (0.66%)), smoking status (never, former, light smoker: ≤15 cigarettes/day, medium smoker: 16–29 cigarettes/day, heavy smoker: ≥30 cigarettes/day, or missing/unknown (0.23%)), alcohol consumption (non-drinkers, <1, 1–9.99, 10–19.99, ≥20 grams/day, or unknown/missing (0.36%)), ethnicity (White, mixed race or other, Asian or British Asian, Black or Black British, or missing/unknown (0.33%)), diabetes (yes, no, or unknown (0.15%)), body mass index (BMI; <20, 20–22.49, 22.5–24.99, 25.0–27.49, 27.5–29.99, 30–32.49, 32.5–34.99, ≥35 kg/m^2^, or unknown/missing (0.20%)), and total energy intake measured from the 24-h dietary assessments (sex-specific quintiles).

For colorectal cancer analyses, models were also adjusted for red and processed meat intake (<2 times per week, 2–2.99 times per week, 3–3.99 times per week and ≥4 times per week, or unknown (0.41%) estimated from the recruitment touchscreen questionnaire as meat intake was infrequently reported in the 24-h dietary assessments) and women specific covariates: menopausal hormone therapy (MHT) use (never, former, current, or unknown (0.12%)) and menopausal status (premenopausal, postmenopausal, or unknown (5.42%) assigning men to a separate category). For breast cancer analyses, models were additionally adjusted for MHT use (same as above), oral contraceptive use (never, former, current, or unknown (0.16%)), age at menarche (≤12 years old, 13 years, ≥14 years, or unknown (21.2%)), parity and age at first birth (nulliparous, 1–2 children <25 years old, ≥3 children <25 years old, 1–2 children 25–29.9 years old, ≥3 children 25–29.9 years old, 1–2 children ≥30 years old, ≥3 children ≥30 years old, or unknown/missing (0.06%)), and menopausal status and BMI (six categories: premenopausal: <25, 25–29.99, ≥30 kg/m^2^ and postmenopausal: <25, 25–29.99, ≥30 kg/m^2^ or unknown (5.42%)). For prostate cancer analyses, models were also adjusted for marital status (not living with a partner, living with a partner) [37]. To assess how the addition of confounders changed the association of dietary exposure of interest with specific cancer sites, χ^2^ statistics and *p*-values for including intake of total protein or dairy protein or dairy sources per 2.5% energy increase in the model were estimated using likelihood ratio tests (LRTs) comparing to a model without percentage of energy from protein or dairy protein in the model [38]. This was similarly done for dietary calcium intake, modelled as a 300 mg/day increment. LRT for departures from linearity were also estimated by comparing models with the dietary exposure of interest modelled as an incremental increase (per 2.5% energy intake/day for total protein, total dairy protein, and protein from individual dairy sources, and per 300 mg/day for calcium) to models where the dietary exposure was modelled as quartiles or using restricted cubic splines (with four spline knots), and no evidence of non-linearity was observed (data not presented). To assess the potential mediating role of IGF-I, we repeated the main multivariable-adjusted Cox regression models with further adjustment for serum IGF-I concentrations (quintiles) measured at recruitment. Assessment of the proportional hazards assumption was evaluated using Schoenfeld residuals and no violation of the assumption was observed (*p* > 0.05).

In additional analyses, we assessed the associations of intakes of total g/day of all dairy products, milk, and cheese with risks of colorectal, breast, and prostate cancer to compare results to previous meta-analyses [2–7]. We additionally conducted risk analyses looking at a 200 g/day increment in dairy products intake, a 200 g/day increment in milk intake, and a 50 g/day increment in cheese intake.

### Subgroup and sensitivity analyses

For subgroup analyses, we assessed heterogeneity by BMI (~median; <27 and ≥27 kg/m^2^), alcohol intake (<10 g/day and ≥10 g/day), and smoking status (ever and never) by using a LRT comparing the main model to a model including an interaction term between dietary intake of protein, dairy protein, or dietary calcium and the subgroup of interest. For colorectal cancer, we further assessed heterogeneity by sex using a LRT. For breast cancer, we assessed heterogeneity by menopausal status by separating follow-up time at age 55 years and their defined menopause status at recruitment so women who were premenopausal at recruitment would change into the postmenopausal risk set at 55 years of age. If a woman was defined as postmenopausal at recruitment, she remained in the postmenopausal risk set regardless of age. We further explored if associations varied by tumour site for colorectal cancer (colon or rectal). In 68 instances, the diagnosis of colon and rectal cancer coincided, and these participants were removed from the subgroup analyses by tumour site. For heterogeneity by tumour site, we stratified Cox models using a competing risks approach [39] and compared the risk coefficients and standard errors of protein intake modelled as a 2.5% energy increment and dietary calcium modelled as a 300 mg/day increment using colon cancer and rectal cancer as separate outcomes.

In sensitivity analyses, we further adjusted for other components of diet including total fibre intake (sex specific quintiles from ≥2 24-h dietary assessments) and fruit and vegetable intake (quintiles derived from ≥2 24-h dietary assessment) as well as red and processed meat intake (touchscreen questionnaire) for breast and prostate cancer analyses. Moreover, we restricted analyses to participants who completed a minimum of three 24-h dietary assessments to reduce random measurement error in the estimation of intake of all dairy protein and dairy protein sources. Finally, to assess for reverse causality, we excluded the first 2 years of follow-up.

All analyses were conducted using Stata version 17.0 (Stata Corp, TX, United States). *P*-values were two-sided with *p* < 0.05 being considered statistically significant.

## Results

Over a median of 9.4 years of follow-up (IQR: 9.3–9.8 years), 1193 incident colorectal cancer, 2024 breast cancer and 2422 prostate cancer cases were observed. Table 1 presents baseline characteristics for the highest and lowest quartile of percentage of energy from protein from all dairy sources, milk, and cheese and total dietary calcium intake. Supplementary Tables 1 and 2 present the baseline characteristics across highest and lowest quartiles of protein from all dairy products, milk, cheese, and total dietary calcium separately for men and women, while Supplementary Table 3 presents the mean and standard deviation intakes of protein from all dairy products, milk, cheese and total dietary calcium by quartiles and separately for men and women. A total of 670 participants (0.6%) reported consuming no dairy products across all completed 24-h dietary assessments. Participants in the highest quartile of protein from all dairy products and milk protein intake were more likely to be women, older, be never smokers, consume less alcohol (~8 g/day less), be of White ethnicity, and report a lower energy intake than those in the lowest quartile of intake. Participants in the highest quartile of cheese protein intake were less physically active and more likely to have a university/college degree than those in the lowest quartile. Supplementary Fig. 3 presents the average grams of protein consumed from each source (all dairy products, milk, and cheese). Most of the protein from dairy products came from milk protein, followed by protein from cheese (Supplementary Fig. 3). Total dietary calcium intake was highly correlated with total dairy protein intake (*r* = 0.80) and calcium from dairy products contributed to 45.7% of dietary calcium intake.

**Table 1:** Baseline characteristics by lowest and highest quartile of percentage of energy intake from protein from all dairy products, milk, and cheese.

	Protein from dairy products		Protein from milk		Protein from cheese		Total dietary calcium	
	Q1	Q4	Q1	Q4	Q1	Q4	Q1	Q4
Number of participants	28,555	28,554	28,555	28,554	30,958	28,554	28,555	28,554
Sex – Female, *N* (%)	13,409 (47.0%)	18,423 (64.5%)	15,688 (54.9%)	17,532 (61.4%)	16,162 (52.2%)	16,468 (57.7%)	17,749 (62.2%)	12,990 (45.5%)
Age at recruitment - years	55.0 (8.0)	56.6 (7.6)	55.2 (8.0)	56.6 (7.6)	55.7 (7.8)	55.8 (7.8)	55.4 (7.8)	56.2 (7.9)
Body mass index – kg/m^2^	26.9 (4.7)	26.6 (4.6)	26.7 (4.8)	26.6 (4.5)	27.0 (4.6)	26.5 (4.6)	26.9 (4.7)	26.6 (4.5)
Height – centimetres	170.3 (9.2)	168.1 (8.9)	169.5 (9.2)	168.3 (9.0)	169.3 (9.2)	169.4 (9.1)	167.7 (8.9)	171.4 (9.2)
Physical activity - High, *N* (%)	4944 (17.3%)	4863 (17.0%)	5127 (18.0%)	4758 (16.7%)	5375 (17.4%)	4796 (16.8%)	4453 (15.6%)	5635 (19.7%)
Townsend deprivation index, *N* (%)								
Q1 - Most affluent	5745 (20.1%)	6512 (22.8%)	5468 (19.1%)	6792 (23.8%)	6716 (21.7%)	6073 (21.3%)	5795 (20.3%)	6451 (22.6%)
Q5 - Most deprived	5606 (19.6%)	6195 (21.7%)	5447 (19.1%)	6457 (22.6%)	6522 (21.1%)	5798 (20.3%)	5648 (19.8%)	6185 (21.7%)
Paid employment, *N* (%)	18875 (66.1%)	16780 (58.8%)	18651 (65.3%)	16691 (58.5%)	19345 (62.5%)	18014 (63.1%)	18485 (64.7%)	17284 (60.5%)
University/college degree, *N* (%)	20793 (72.8%)	20922 (73.3%)	21437 (75.1%)	20365 (71.3%)	21288 (68.8%)	21932 (76.8%)	20215 (70.8%)	21680 (75.9%)
White ethnicity, *N* (%)	26923 (94.3%)	27922 (97.8%)	26908 (94.2%)	28021 (98.1%)	29331 (94.7%)	27826 (97.5%)	26956 (94.4%)	27827 (97.5%)
Smoking - Never, *N* (%)	15390 (53.9%)	17192 (60.2%)	15499 (54.3%)	17478 (61.2%)	18010 (58.2%)	16055 (56.2%)	15710 (55.0%)	16992 (59.5%)
Diabetes - Yes, *N* (%)	1163 (4.1%)	1040 (3.6%)	987 (3.5%)	1153 (4.0%)	1379 (4.5%)	1072 (3.8%)	1115 (3.9%)	1082 (3.8%)
Living with a partner, *N* (%)	20932 (73.3%)	20914 (73.2%)	20540 (71.9%)	21502 (75.3%)	22647 (73.2%)	20894 (73.2%)	20783 (72.8%)	21235 (74.4%)
Women specific variables								
Age at menarche - years	12.6 (2.7)	12.5 (2.6)	12.5 (2.7)	12.6 (2.6)	12.6 (2.7)	12.5 (2.7)	12.6 (2.7)	12.6 (2.6)
Postmenopausal at recruitment, *N* (%)	8308 (62.0%)	13272 (72.1%)	10119 (64.5%)	12621 (72.0%)	10931 (67.6%)	11115 (67.5%)	11663 (65.7%)	9052 (69.7%)
Menopausal hormone therapy use - current, *N* (%)	1109 (8.3%)	1455 (7.9%)	1334 (8.5%)	1344 (7.7%)	1365 (8.4%)	1301 (7.9%)	1597 (9.0%)	984 (7.6%)
Oral contraceptive use - current, *N* (%)	391 (2.9%)	352 (1.9%)	458 (2.9%)	350 (2.0%)	387 (2.4%)	410 (2.5%)	508 (2.9%)	283 (2.2%)
Nulliparous, *N* (%)	3862 (13.5%)	2371 (8.3%)	3483 (12.2%)	2441 (8.5%)	3595 (11.6%)	2942 (10.3%)	2741 (9.6%)	3646 (12.8%)
Diet variables								
Alcohol intake - g/day	20.4 (20.3)	12.2 (12.8)	18.9 (20.3)	12.2 (12.6)	16.2 (18.0)	15.8 (16.2)	17.4 (17.9)	15.2 (15.6)
Red and processed meat intake – times/week	3.6 (2.3)	3.2 (2.1)	3.3 (2.3)	3.4 (2.0)	3.6 (2.2)	3.2 (2.2)	3.4 (2.2)	3.4 (2.3)
Vegetable and fruit intake - g/day	374.5 (235.5)	395.4 (224.0)	409.6 (249.6)	365.7 (205.9)	376.1 (235.3)	388.4 (223.0)	332.3 (201.5)	436.8 (251.8)
Total dairy product intake - g/day	169.8 (95.6)	470.5 (175.0)	178.5 (126.5)	456.0 (160.9)	301.7 (172.5)	334.8 (165.5)	184.0 (97.7)	472.1 (182.3)
Total milk intake - g/day	104.9 (84.0)	279.5 (134.1)	54.6 (48.9)	332.4 (106.3)	195.9 (125.3)	192.3 (121.0)	116.1 (84.5)	280.4 (139.0)
Total cheese intake - g/day	7.0 (9.4)	29.7 (22.2)	19.1 (19.8)	15.0 (15.9)	0.00 (0.00)	40.4 (17.4)	8.8 (11.2)	28.2 (22.2)
Total protein intake - % of energy	15.3 (3.1)	16.9 (3.1)	15.4 (3.2)	17.0 (3.0)	16.2 (3.4)	16.1 (3.1)	1.0 (0.8)	1.7 (0.9)
Dairy product protein - % of energy	1.3 (0.5)	4.8 (1.0)	1.9 (1.2)	4.1 (1.3)	2.2 (1.2)	4.1 (1.3)	2.0 (1.1)	3.8 (1.4)
Milk protein - % of energy	0.7 (0.5)	2.1 (1.0)	0.3 (0.3)	2.5 (0.7)	1.4 (0.9)	1.3 (0.9)	1.0 (0.8)	1.7 (0.9)
Cheese protein - % of energy	0.3 (0.4)	1.5 (1.1)	0.9 (0.9)	0.8 (0.8)	0.00 (0.00)	2.0 (0.7)	0.5 (0.7)	1.2 (1.0)
Total carbohydrate intake - % of energy	48.3 (7.8)	50.3 (7.3)	48.1 (8.2)	51.2 (6.8)	50.8 (7.6)	47.7 (7.5)	47.9 (8.5)	50.5 (6.6)
Total fat intake - % of energy	31.4 (6.0)	31.3 (5.9)	32.2 (6.2)	30.3 (5.5)	30.0 (5.9)	33.2 (5.8)	31.1 (6.3)	32.0 (5.4)
Total fibre intake - g/day	18.0 (6.1)	17.4 (5.5)	18.4 (6.3)	16.9 (5.2)	17.3 (5.9)	17.9 (5.7)	14.6 (4.6)	21.2 (6.0)
Total dietary calcium intake – mg/day	784 (233)	1169 (287)	849 (273)	1070 (286)	853 (260)	1105 (295)	644 (105)	1359 (196)
Total energy intake - kJ/day	8860 (2032)	8094 (1816)	8775 (2049)	7847 (1690)	8224 (1937)	8607 (1951)	7199 (1476)	10,117 (1850)

Values are mean (SD) unless otherwise indicated.

*g/day* grams per day, *kg/m*^*2*^ kilograms per metre squared, *kJ/day* kilojoules per day, *mg/day* milligrams per day, *N* Number of participants, *Q* quantile, SD standard deviation.

**Fig. 1:**
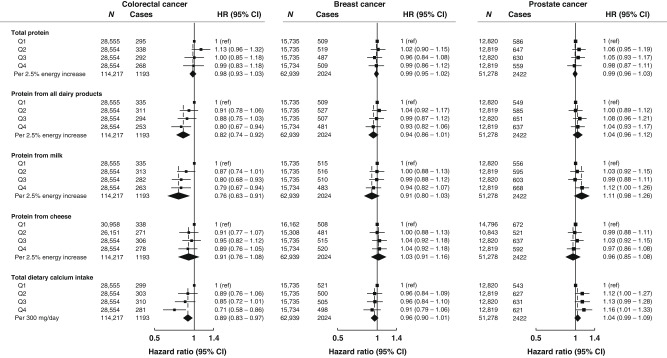
Multivariable-adjusted hazard ratios (95% CI) for intakes of total protein, protein from all dairy products and dairy sources, and dietary calcium and colorectal, breast, and prostate cancer risk. All models used age as the underlying time variable, were stratified by age groups at recruitment, and were further adjusted for height, physical activity, Townsend deprivation index, education, employment status, smoking status, alcohol intake, ethnicity, diagnosis of diabetes, BMI, energy intake. For colorectal cancer analyses: all models were stratified by sex and further adjusted for menopausal status (women only), menopausal hormone therapy use (women only), red and processed meat intake, and non-steroidal anti-inflammatory drug use. For breast cancer analyses: models were further adjusted for menopausal hormone therapy use, oral contraceptive use, parity and age at first birth, age at menarche, BMI and menopausal status. For prostate cancer analyses: models were further adjusted for marital status. Abbreviations: BMI body mass index, CI confidence interval, HR hazard ratio, N number of participants, Q quantile, ref reference group.

Fully adjusted models presenting the HR and 95% CI for total protein, all dairy protein, milk protein, cheese protein, and total dietary calcium intakes and risks of colorectal, breast, and prostate cancer are presented in Fig. 1, while minimally adjusted models with sequential adjustment of potential confounders are shown in Supplementary Tables 4–6. After adjustment for potential confounders, intakes of protein from all dairy sources and from milk were inversely associated with colorectal cancer risk (HR _Q4 vs. Q1_: 0.80, 95% CI: 0.67–0.94, *p*-_trend_ = 0.001 and 0.79, 0.67–0.94, *p*-_trend_ = 0.003, respectively; Fig. 1) whereas no statistically significant association was observed for intake of total protein or protein from cheese. Total calcium intake was also inversely associated with colorectal cancer risk (HR _Q4 vs. Q1_: 0.71, 95% CI: 0.58–0.97, *p*-trend = 0.004). For breast cancer risk, we did not observe evidence of an association across intakes of total protein, protein from all dairy products or dairy sources, or total dietary calcium intake (Fig. 1). For prostate cancer, no clear associations were observed for intakes of total protein, protein from all dairy products or cheese. However, a positive association was suggested for men in the highest quartile of protein from milk and dietary calcium intake in comparison to the lowest quartile (HR_Q4 vs Q1_: 1.12, 95% CI: 1.00–1.26; 1.16, 1.01–1.33, respectively), although these associations were not statistically significant when milk protein or calcium intake were modelled as continuous variables (HR_per 2.5% energy increment_: 1.11, 95% CI: 0.98–1.26; HR_per 300 mg/day_: 1.04, 0.99–1.09, respectively; Fig. 1). Moreover, when IGF-I concentrations measured at recruitment were added into the multivariable adjusted models, associations remained largely unchanged (Table 2).

**Table 2:** Hazard ratios (95% CI) for intake of total protein, protein from dairy products and dairy sources, and calcium with colorectal, breast, and prostate cancer with additional adjustments for circulating insulin-like growth factor-I (IGF-I).

Colorectal cancer	Intakes of total protein, protein from dairy products, and calcium						
	Q1	Q2	Q3	Q4	Per 2.5% energy intake or 300 mg/day^a^	χ^2^	*P-trend*
Total protein intake							
Fully adjusted model	1 (ref)	1.13 (0.96–1.32)	1.00 (0.85–1.18)	0.99 (0.83–1.18)	0.98 (0.93–1.03)	0.79	0.37
Fully adjusted model + IGF-I concentrations	1 (ref)	1.13 (0.96–1.32)	1.00 (0.85–1.18)	0.99 (0.83–1.18)	0.98 (0.93–1.03)	0.78	0.38
Protein from dairy products							
Fully adjusted model	1 (ref)	0.91 (0.78–1.06)	0.88 (0.75–1.03)	**0.80 (0.67–0.94)**	**0.82 (0.74–0.92)**	11.61	0.001
Fully adjusted model + IGF-I concentrations	1 (ref)	0.91 (0.78–1.06)	0.88 (0.75–1.03)	**0.80 (0.67–0.94)**	**0.82 (0.74–0.92)**	11.58	0.001
Protein from milk							
Fully adjusted model	1 (ref)	0.87 (0.74–1.01)	**0.80 (0.68–0.93)**	**0.79 (0.67–0.94)**	**0.76 (0.63–0.91)**	9.01	0.003
Fully adjusted model + IGF-I concentrations	1 (ref)	0.87 (0.74–1.01)	**0.80 (0.68–0.93)**	**0.79 (0.67–0.94)**	**0.76 (0.63–0.91)**	9.02	0.003
Protein from cheese							
Fully adjusted model	1 (ref)	0.91 (0.77–1.07)	0.95 (0.82–1.12)	0.89 (0.76–1.05)	0.91 (0.76–1.08)	1.25	0.26
Fully adjusted model + IGF-I concentrations	1 (ref)	0.91 (0.77–1.07)	0.96 (0.82–1.12)	0.89 (0.76–1.05)	0.91 (0.76–1.08)	1.24	0.26
Total dietary calcium							
Fully adjusted model	1 (ref)	0.89 (0.76–1.06)	0.85 (0.72–1.01)	**0.71 (0.58–0.86)**	**0.89 (0.83–0.97)**	8.39	0.004
Fully adjusted model + IGF-I concentrations	1 (ref)	0.89 (0.76–1.06)	0.85 (0.72–1.01)	**0.71 (0.58–0.86)**	**0.89 (0.83–0.97)**	8.38	0.004

Breast cancer	Intakes of total protein, protein from dairy products, and calcium						
	Q1	Q2	Q3	Q4	Per 2.5% energy intake or 300 mg/day^a^	χ^2^	*P-trend*
Total protein intake							
Fully adjusted model	1 (ref)	1.02 (0.90–1.15)	0.96 (0.84–1.08)	0.99 (0.86–1.12)	0.99 (0.95–1.02)	0.47	0.49
Fully adjusted model + IGF-I concentrations	1 (ref)	1.01 (0.89–1.14)	0.95 (0.84–1.08)	0.97 (0.85–1.11)	0.98 (0.95–1.02)	0.77	0.38
Protein from dairy products							
Fully adjusted model	1 (ref)	1.04 (0.92–1.17)	0.99 (0.87–1.12)	0.93 (0.82–1.06)	0.94 (0.86–1.01)	2.72	0.10
Fully adjusted model + IGF-I concentrations	1 (ref)	1.04 (0.92–1.17)	0.99 (0.87–1.12)	0.93 (0.82–1.05)	0.93 (0.86–1.01)	3.02	0.08
Protein from milk							
Fully adjusted model	1 (ref)	1.00 (0.88–1.13)	0.99 (0.88–1.12)	0.94 (0.83–1.07)	0.91 (0.80–1.03)	2.09	0.15
Fully adjusted model + IGF-I concentrations	1 (ref)	0.99 (0.88–1.12)	0.99 (0.88–1.12)	0.92 (0.81–1.05)	0.90 (0.79–1.02)	2.77	0.10
Protein from cheese							
Fully adjusted model	1 (ref)	1.00 (0.88–1.14)	1.04 (0.92–1.18)	1.04 (0.92–1.18)	1.03 (0.91–1.16)	0.21	0.65
Fully adjusted model + IGF-I concentrations	1 (ref)	0.99 (0.87–1.13)	1.04 (0.92–1.18)	1.04 (0.92–1.18)	1.03 (0.91–1.17)	0.27	0.60
Total dietary calcium							
Fully adjusted model	1 (ref)	0.96 (0.84–1.09)	0.96 (0.84–1.10)	0.91 (0.79–1.06)	0.96 (0.90–1.01)	2.22	0.14
Fully adjusted model + IGF-I concentrations	1 (ref)	0.95 (0.84–1.08)	0.96 (0.84–1.09)	0.90 (0.78–1.05)	0.95 (0.89–1.01)	2.79	0.09

Prostate cancer	Intakes of total protein, protein from dairy products, and calcium						
	Q1	Q2	Q3	Q4	Per 2.5% energy intake or 300 mg/day^a^	χ^2^	*P-trend*
Total protein intake							
Fully adjusted model	1 (ref)	1.06 (0.95–1.19)	1.05 (0.93–1.17)	0.98 (0.87–1.11)	0.99 (0.96–1.03)	0.18	0.68
Fully adjusted model + IGF-I concentrations	1 (ref)	1.06 (0.95–1.19)	1.04 (0.93–1.17)	0.97 (0.86–1.10)	0.99 (0.95–1.03)	0.30	0.59
Protein from dairy products							
Fully adjusted model	1 (ref)	1.00 (0.89–1.12)	1.08 (0.96–1.21)	1.04 (0.93–1.17)	1.04 (0.96–1.12)	0.85	0.36
Fully adjusted model + IGF-I concentrations	1 (ref)	1.00 (0.89–1.12)	1.07 (0.96–1.20)	1.03 (0.92–1.16)	1.03 (0.96–1.12)	0.67	0.41
Protein from milk							
Fully adjusted model	1 (ref)	1.03 (0.92–1.15)	0.99 (0.88–1.11)	**1.12 (1.00–1.26)**	1.11 (0.98–1.26)	2.74	0.10
Fully adjusted model + IGF-I concentrations	1 (ref)	1.03 (0.91–1.15)	0.99 (0.88–1.11)	1.11 (0.99–1.25)	1.11 (0.97–1.26)	2.40	0.12
Protein from cheese							
Fully adjusted model	1 (ref)	0.99 (0.88–1.11)	1.03 (0.92–1.15)	0.97 (0.86–1.08)	0.96 (0.85–1.08)	0.52	0.47
Fully adjusted model + IGF-I concentrations	1 (ref)	0.99 (0.88–1.11)	1.03 (0.92–1.15)	0.97 (0.86–1.08)	0.96 (0.85–1.08)	0.53	0.47
Total dietary calcium							
Fully adjusted model	1 (ref)	**1.12 (1.00–1.27)**	1.13 (0.99–1.28)	**1.16 (1.01–1.33)**	1.04 (0.99–1.09)	2.05	0.15
Fully adjusted model + IGF-I concentrations	1 (ref)	1.12 (0.99–1.26)	1.12 (0.99–1.27)	**1.15 (1.00–1.32)**	1.03 (0.98–1.09)	1.67	0.20

Estimates in bold indicate statistically significant results.

All models used age as the underlying time variable, were stratified by age groups at recruitment, and further adjusted for height, physical activity, Townsend deprivation index, education, employment status, smoking status, alcohol intake, ethnicity, diagnosis of diabetes, BMI, energy intake.

For colorectal cancer analyses: all models were stratified by sex and further adjusted for menopausal status (women only), menopausal hormone therapy use (women only), red and processed meat intake, and non-steroidal anti-inflammatory drug use.

For breast cancer analyses: models were further adjusted for menopausal hormone therapy use, oral contraceptive use, parity and age at first birth, age at menarche, BMI and menopausal status.

For prostate cancer analyses: models were further adjusted for marital status.

Fully adjusted model + IGF-I concentrations were further adjusted serum circulating insulin-like growth factor-I concentrations measured at recruitment.

*CI* confidence intervals, *BMI* body mass index, *IGF-I* insulin-like growth factor-I, *Q* quantile, *ref* reference.

^a^Total protein and protein from dairy sources are modelled as a 2.5% energy increase whereas total dietary calcium intake is modelled as a 300 mg/day increase.

In additional analyses on the associations of intake of total g/day (not only from protein) of all dairy products, milk, and cheese with colorectal, breast, and prostate cancer risk, the associations were generally similar to those from analyses looking at protein from all dairy and dairy sources expressed as a 2.5% energy increment. However, there was a suggestion of an inverse association between g/day of total dairy intake and breast cancer risk when comparing the highest quartile to the lowest quartile of intake (HR_Q4 vs Q1_: 0.88, 0.77–1.00), although there was no statistically significant trend (HR_per 200 g/day of increase_: 0.90, 0.81–1.01); Supplementary Fig. 4).

### Subgroup and sensitivity analyses

For breast cancer risk, there was some evidence of heterogeneity for total protein intake where premenopausal women had a higher risk of breast cancer with higher protein intake (HR_per 2.5% energy_: 1.13, 1.03–1.23) whereas no evidence of an association was observed for postmenopausal breast cancer (0.96, 0.92–1.00; *p*-_het_ = 0.03; Supplementary Fig. 5). For colorectal cancer risk, evidence of heterogeneity was observed by alcohol intake for protein from all dairy products; an inverse association was only observed for those consuming ≥10 g/day of alcohol (HR_per 2.5% energy_: 0.73, 0.63–0.86) but no evidence of an association was observed for those consuming <10 g/day of alcohol (0.94, 0.80–1.10; *p*-_het_ = 0.026; Supplementary Fig. 6). We observed similar evidence of heterogeneity for intake of protein from milk by alcohol intake, and also by smoking status; for individuals who consumed ≥10 g/day of alcohol and for those who were ever smokers, intake of protein from milk was inversely associated with colorectal cancer risk (HR_per 2.5% energy_: 0.61, 0.47–0.79; *p*-_het_ = 0.022 and 0.60, 0.46–0.78; *p*-_het_ = 0.009, respectively) but no evidence of an association was observed for those who consumed <10 g/day of alcohol or were never smokers (Supplementary Fig. 7). For breast and prostate cancer analyses, no evidence of heterogeneity by alcohol, smoking status, BMI were observed (Supplementary Figs 5–8). No differences in risk were observed between total protein, dairy protein, or total dietary calcium intake and risk of colon or rectal cancer (Supplementary Table 7).

When we further adjusted for other dietary factors in sensitivity analyses, associations remained largely the same with the exception that the association for protein from milk and prostate cancer risk was attenuated and the HR for Q4 vs. Q1 was no longer significant (1.10, 0.98–1.24; Supplementary Table 8), which was attributed to red and processed meat in the model being positively associated with prostate cancer risk and with milk intake. In analyses restricted to participants who completed a minimum of three 24-h dietary assessments, associations remained largely unchanged but with wider confidence intervals probably due to the loss of participants and cases (Supplementary Table 9). No material differences in associations were observed in analyses removing the first two years of follow-up (Supplementary Table 10).

## Discussion

In this analysis using data from the UK Biobank study, higher intakes of protein from all dairy products and milk, as well as total dietary calcium intake, were associated with a lower risk of colorectal cancer. There was some evidence that men with high compared to low intakes of protein from milk and total dietary calcium may have a slightly higher risk of being diagnosed with prostate cancer, although there was no evidence of a linear trend of increasing risk with higher milk protein or dietary calcium intake. Moreover, we did not observe evidence of an association between protein or dietary calcium intake and breast cancer risk in women.

### Colorectal cancer

To the best of our knowledge, only two previous prospective studies have looked at the association of dairy protein and its sources with colorectal cancer risk [11, 12], with both suggesting an inverse association with risk. Milk protein intake was the major contributor to all dairy protein in this analysis (~50%) and the observed inverse association for dairy protein intake is probably driven by milk consumption.

In the most recent WCRF meta-analysis (from 2017) including 14 prospective studies, intake of total grams of all dairy products combined was associated with a 13% lower risk of colorectal cancer risk per 400 g/day intake [2, 3]. Similarly, this meta-analysis found that in 13 prospective studies milk intake was associated with a 6% lower risk of colorectal cancer per 200 g/day increment, whereas for cheese intake a non-significant inverse association was observed [2, 3]. For comparison, our study observed a 12% lower risk of colorectal cancer per 200 g/day of total dairy intake, and a 13% lower risk of colorectal cancer per 200 g/day intake of milk. Moreover, in MR analyses utilising the lactase persistence gene as a proxy for milk intake, which has been shown in a European population to predict a 17.1 g/day per allele difference in milk intake [40], a lower risk of colorectal cancer was observed among those who had the lactase persistence genotype and thus were assumed to consume more milk (odds ratio per allele: 0.95, 95% CI: 0.91–0.99) [41].

It is likely that the protein content in dairy products or milk is not the driving force for the observed lower risk of colorectal cancer, as there are some other compounds and minerals in milk, such as calcium, for which there is stronger evidence that they may reduce the risk of colorectal cancer [42]. In the current analysis dietary calcium intake, which was largely from dairy products, was inversely associated with colorectal cancer risk. Higher calcium intake has been previously shown to be associated with lower colorectal cancer risk in prospective studies [2, 43]. Calcium may protect against colorectal cancer risk directly by increasing intra-luminal apoptosis of colonic epithelium cells [44] as well as locally binding to secondary bile acids [45] and thus inhibiting their potential carcinogenic effect. However, other components of dairy products, such as conjugated linoleic acid and butyric acid, may also be inversely associated with colorectal cancer risk [42, 46], and isolating the impacts of these food components, which are consumed together in milk, is challenging.

In our analyses, we observed heterogeneity by both alcohol intake and smoking status in the association of intake of protein from milk with colorectal cancer risk; the inverse association of milk protein intake with colorectal cancer risk was observed only in individuals who consumed ≥10 g/day of alcohol, or were ever smokers. To our knowledge, previous prospective studies have not explored whether the association of dairy products intake with colorectal cancer risk varies by these lifestyle factors [47–50]. Moreover, in agreement with our study, most previous prospective studies have observed that those who consume higher amounts of dairy products typically consume less alcohol on average [47–51]. This observed heterogeneity could suggest residual confounding by alcohol and smoking, for example if low milk consumers under-report their alcohol consumption more than high milk consumers, or this finding may be due to chance.

### Breast cancer

Intakes of total protein, protein from all dairy products and its sources, and calcium were not associated with breast cancer risk in this study. To the best of our knowledge, only one previous prospective study has looked at the association of dairy protein; in a recent prospective analysis of 700,000 postmenopausal UK women from the Million Women Study (published after the most recent WCRF meta-analysis from 2017) [5], which included 29,000 incident breast cancer cases diagnosed over 12 years of follow-up, intakes of total protein, dairy protein, as well as milk, yogurt, or cheese, were not associated with breast cancer risk [9]. However, we did observe evidence of heterogeneity by menopausal status for total protein intake with a higher risk for premenopausal women and not postmenopausal women, although there were few cases of premenopausal breast cancer therefore this finding may be due to chance. When we looked at total grams of all dairy products combined, those in the highest quartile of intake had a suggestive lower risk of breast cancer than those in the lowest quartile, although there was no significant trend. Results from previous meta-analyses and recent studies have also been inconsistent; the 2017 WCRF meta-analysis, including seven prospective studies, suggested that a higher intake of dairy products (per 200 g/day) was associated with a 5% lower risk of premenopausal breast cancer, and no association was found with postmenopausal breast cancer [4]. After this meta-analysis was published, two prospective studies including 2582 and 1057 breast cancer cases suggested a positive association between dairy intake and breast cancer risk [52–54]. In 2021, a pooled analysis of 21 cohort studies with individual level data including 37,000 breast cancer cases found a 5% lower risk of total breast cancer for participants in the highest category of milk intake in comparison to lowest [55]. However, MR analyses using the lactase persistence gene suggest no association between milk intake and overall breast cancer risk [41]. In line with our findings, no association was observed between the highest quintile versus the lowest quintile of dietary calcium intake and breast cancer risk in a pooled analysis of 21 cohort studies [55].

### Prostate cancer

We found a suggestive positive association between intake of protein from milk and prostate cancer risk. However, this association was attenuated when other dietary components were adjusted for in multivariable models. We also observed a similar positive association between total calcium intake and prostate cancer risk. No other associations between intakes of protein from all dairy products or cheese and prostate cancer risk were observed. To the best of our knowledge, only three previous prospective studies have looked at the association of protein from dairy products and prostate cancer risk; two studies including men in the European Prospective Investigation into Cancer and Nutrition (EPIC) [8, 13] where the most recent analysis, including 131,425 men of whom 6939 men developed prostate cancer, found a higher risk among the men in the top three fifths in comparison to the lowest fifth of intake of dairy protein and yogurt protein [13]. The third study to assess this association was a nested case-control study in the Breast and Prostate Cancer Cohort Consortium, which included 4815 cases and 4671 controls (including 728 cases from EPIC) and found no association between dairy protein intake and prostate cancer risk [10]. When we looked at total grams of all dairy products, milk, and cheese we found no association with prostate cancer risk, whereas the latest WCRF meta-analysis of prospective studies from 2014 found that intakes of total grams of all dairy products, milk, and cheese were all associated with a higher risk of prostate cancer [6, 7, 56]. MR analyses using the lactase persistence gene as a proxy for milk intake have generally found no association [41]; however, using data from a Finnish consortium (3282 prostate cancer cases and 55,968 controls), a population in which milk intake is typically high, the MR analysis showed a significant positive association between genetically predicted milk intake and prostate cancer risk [41]. In these analyses we did not find a significant linear association between protein from milk or dietary calcium intake and prostate cancer risk, and although a linear trend is not mandated, there was also no evidence of non-linearity, and future research is needed to explore possible trends across intakes.

The possible positive association between protein from milk and prostate cancer risk has been hypothesised to be mediated by circulating IGF-I concentrations, which have been shown to be associated with both higher dairy protein intake and prostate cancer risk [15, 17, 18], and possibly prostate cancer mortality [21]. However, when we added circulating IGF-I concentrations measured at baseline (before dietary assessments) into multivariable-adjusted models, the association of protein from milk with prostate cancer risk was only slightly attenuated suggesting that circulating IGF-I concentrations may not explain this association, although the use of a single measure of IGF-I for each person is a limitation. Due to its high content of branched chain amino acids, particularly leucine [57], milk intake may also activate the mechanistic target of rapamycin (mTOR) pathway and mTOR complex 1 which has been implicated in carcinogenesis of the prostate [58]. However, we also observed a positive association between total calcium intake and prostate cancer risk, and some evidence suggests that higher calcium intake may downregulate 1,25-dihydroxyvitamin D concentrations and high 1,25-dihydroxyvitamin D concentrations may inhibit cellular proliferation of the prostate [59, 60]. However a recent collaborative analysis of 3 studies has suggested that higher circulating 1,25-dihydroxyvitamin D concentrations were not associated with prostate cancer risk [61]. The observed results for milk protein may also be subject to detection bias if men who consume more milk are more likely to have a prostate-specific antigen (PSA) test and therefore a prostate cancer diagnosis. Moreover, in our current analysis, we assessed risk of total prostate cancer because data on tumour subtypes were not available; however, risk factors for prostate cancer may vary by tumour characteristics [62] and further research is needed to understand these mechanisms and how they influence prostate carcinogenesis, particularly for aggressive and lethal prostate cancer.

### Possible impact of dairy protein on cancer through IGF-I concentrations

Although dairy protein intake, and more specifically milk protein intake, has been associated with higher circulating IGF-I concentrations [14, 15], the magnitude of this relationship and therefore any impact on cancer risk may not be large. In previous cross-sectional analyses in UK Biobank, a 2.5% higher energy intake from milk protein was associated with a 1.20 nmol/L higher circulating IGF-I concentration [15]. In prospective analyses, higher IGF-I concentrations (per 5 nmol/L) have been associated with ~9–11% higher risks of colorectal, breast, and prostate cancer [19–21]. Similar estimates have been observed utilising MR analyses [19, 20], with the exception that a 5 nmol/L genetically predicted higher IGF-I concentration has been associated with a 34% higher risk of prostate cancer when using a *cis*-SNP, although CIs were wide [21]. Based on these estimates, we might expect to see a ~2% higher risk for both colorectal and breast cancer and a ~2–8% higher risk of prostate cancer per 2.5% higher energy intake from milk protein; although it should be noted that due to measurement error in dietary assessments the true risks might be larger. While protein from dairy products may elevate IGF-I concentrations, other components of dairy products and milk may protect against the risk of cancer at some sites; therefore, further research examining other mechanisms and compounds present in dairy products is needed to better understand how intake of dairy products may influence cancer risk.

### Strengths and limitations

The strengths of these analyses include the prospective nature of the study, detailed estimates of intakes of protein, dairy protein, and dietary calcium obtained from 2 to 5 24-h dietary assessments which reduces random measurement error, and use of record linkage limiting loss to follow-up and outcome misclassification. The detailed dietary assessment allowed for the examination of two specific sources of dairy products in association with common cancers. As well, the UK Biobank collected detailed sociodemographic, anthropometric, and lifestyle information from participants at recruitment, allowing adjustment for these potential confounders and other components of diet in the analyses. Moreover, participants in the UK Biobank also provided a blood sample at recruitment in which IGF-I concentrations were estimated, allowing us to assess how IGF-I concentrations influence the associations between dairy products and risk of IGF-I related cancers.

There are also some limitations to consider. All self-reported dietary intake data are subject to measurement error, and although online 24-h dietary assessments offer advantages, such as quick completion by participants, multiple 24-h dietary assessments are needed to reliably estimate average intakes due to the substantial day-to-day variation. In this sample, approximately 35% of participants completed only two 24-h dietary assessments, which will increase random measurement error thus biasing the estimates towards null and reducing the statistical power of these analyses. This may be particularly important for cheese intake as previous evidence has shown low reproducibility (intra-class correlation = 0.38) comparing the average of two 24-h dietary assessments to two other independent 24-h dietary assessements [63]. However, we did conduct a sensitivity analysis restricting to participants with at least three completed 24-h dietary assessments and saw similar results but with wider confidence intervals due to the loss of participants and cases. We also could not assess associations with all individual dairy sources, such as yogurt, as it was consumed episodically in this sample and therefore a small number of 24-h dietary assessments per person cannot accurately estimate usual intake. We assessed intake of protein from dairy products, as this macronutrient has been previously associated with IGF-I concentrations, but other nutrients found in dairy products are highly correlated and it is therefore difficult in observational studies to separate the associations for different nutrients in these foods. The UK Biobank is also known to be a generally healthy sample of participants, and the subsample of participants in our analyses are likely to be even more health-conscious, therefore the results reported here might not be generalisable to other populations and this also may bias the observed results [64], although estimates may remain in the same direction [65]. As well, some analyses may have insufficient statistical power, particularly for sensitivity and subgroup analyses where the numbers of cases observed were limited. Moreover, some recent evidence has suggested stronger associations with breast and prostate cancer at lower intakes of dairy [52, 54, 66] and although we did not observe this in these analyses, analyses may be underpowered due to limited number of participants reporting this range of intakes. We were also only able to use one measure of IGF-I concentrations in multivariable-adjusted models, therefore use of these measurements as estimates of typical long-term concentrations is subject to error and may underestimate the extent of mediation. Moreover, mediation analysis has key assumptions, such as no unmeasured mediator-outcome confounding or exposure-mediator confounding [67], and some of these assumptions may be invalid if unmeasured confounders were not adjusted for and would therefore influence the observed results. It is possible that associations may differ by different tumour subtypes, but data on tumour subtypes (e.g., hormone receptor status for breast cancer, aggressiveness of tumour for prostate cancer) were not available in the UK Biobank dataset at the time of these analyses. Finally, as with any observational study, the observed associations are subject to residual and unmeasured confounding.

In conclusion, we found that higher intakes of protein from dairy products, and protein from milk, were inversely associated with colorectal cancer risk, although based on previous evidence it is possible that these associations are driven by other components present in dairy products such as calcium, and our analyses also showed that higher dietary calcium intake was inversely associated with colorectal cancer risk. A higher intake of protein from milk and dietary calcium were weakly positively associated with prostate cancer risk, although these associations were only significant when we compared the highest with the lowest quartile and we did not find a significant linear trend. We did not observe evidence of any associations between intakes of protein from dairy products sources and breast cancer risk. Further research is needed on both the IGF pathway and other mechanisms that may account for the possible impacts of dairy products and calcium on cancer risk.

## Supplementary information

Supplementary Materials

## Data Availability

Bona fide researchers can apply to use the UK Biobank dataset by registering and applying at http://ukbiobank.ac.uk/register-apply/.
