# Supplementary material for: Associations of intakes of total protein, protein from dairy sources, and dietary calcium with risks of colorectal, breast, and prostate cancer: a prospective analysis in UK Biobank
Source: Br J Cancer. 2023 Jul 5;129(4):636–47. doi: 10.1038/s41416-023-02339-2 (PMC10421858; doi:10.1038/s41416-023-02339-2)
Supplement: Supplementary file 1 — Supplementary Materials [file 41416_2023_2339_MOESM1_ESM.docx]

**Associations of intake of total protein, protein from dairy sources, and dietary calcium with risks of colorectal, breast, and prostate cancer: a prospective analysis in UK Biobank**

**Authors:** Cody Z. Watling^1^, Rebecca K. Kelly^1^, Yashvee Dunneram^1^, Anika Knuppel^2^, Carmen Piernas^3^, Julie A. Schmidt^4^, Ruth C. Travis^1^, Timothy J. Key^1^, Aurora Perez-Cornago^1^

**Author’s affiliations:**

^1^ Cancer Epidemiology Unit, Nuffield Department of Population Health, University of Oxford, Oxford, United Kingdom.

^2^ MRC Unit of Lifelong Health and Ageing, University College London, London, United Kingdom.

^3^ Nuffield Department of Primary Care, University of Oxford, Oxford, United Kingdom.

^4^ Department of Clinical Epidemiology, Department of Clinical Medicine, Aarhus University and Aarhus University Hospital, Aarhus, Denmark

**Supplementary Methods and Materials**

[Supplementary Methods 4](#_Toc129632764)

[Estimation of dietary intake 4](#_Toc129632765)

[Other dietary variables 5](#_Toc129632766)

[Covariates 6](#_Toc129632767)

[Supplementary Tables 13](#_Toc129632768)

[**Supplementary Table 1.** Baseline characteristics of **women** by lowest and highest quartile of percentage of energy intake from protein from all dairy products, milk, and cheese and total dietary calcium intake 13](#_Toc129632769)

[**Supplementary Table 2.** Baseline characteristics **of men** by lowest and highest quartile of percentage of energy intake from protein from all dairy products, milk, and cheese and total dietary calcium intake. 15](#_Toc129632770)

[**Supplementary Table 3.** Dairy protein intake in grams and percentage of energy per day by quartiles of intake in all participants and separately by males and females. 17](#_Toc129632771)

[**Supplementary Table 4.** Hazard ratios and 95% confidence intervals for minimally adjusted and sequential adjustment models for total protein, protein from all dairy products, milk, and cheese, and total dietary calcium intake and risk of **colorectal cancer.** 19](#_Toc129632772)

[**Supplementary Table 5.** Hazard ratios and 95% confidence intervals for minimally adjusted and sequential adjustment models for total protein, protein from all dairy products, milk, and cheese, and total dietary calcium intake and risk of **breast cancer.** 21](#_Toc129632773)

[**Supplementary Table 6.** Hazard ratios and 95% confidence intervals for minimally adjusted and sequential adjustment models for total protein, protein from all dairy products, milk, and cheese, and total dietary calcium intake and risk of **prostate cancer.** 23](#_Toc129632774)

[**Supplementary Table 7.** Multivariable-adjusted hazard ratios (95% CI) for colon and rectal cancer by intake of total protein, protein from all dairy products, milk, and cheese, and total dietary calcium. 25](#_Toc129632775)

[**Supplementary Table 8.** Hazard ratios (95% CI) for intake of total protein, protein from dairy products and dairy sources, and total dietary calcium with colorectal, breast, and prostate cancer with **additional adjustments for other dietary factors.** 26](#_Toc129632776)

[**Supplementary Table 9.** Hazard ratios (95% CI) for intake of total protein, protein from dairy and dairy sources, and total dietary calcium with risk of colorectal, breast, and prostate cancer for participants who completed a minimum of **three 24-hour dietary assessments.** 29](#_Toc129632777)

[**Supplementary Table 10.** Hazard ratios (95% CI) for intake of total protein, protein from dairy products and dairy sources, and total dietary calcium with risk of colorectal, breast, and prostate cancer **removing the first two years of follow-up.** 30](#_Toc129632778)

[Supplementary Figures 32](#_Toc129632779)

[**Supplementary Figure 1.** Dietary assessment for participants in the UK Biobank over time. 32](#_Toc129632780)

[**Supplementary Figure 2.** Flow diagram showing eligible participants for this study. 33](#_Toc129632781)

[**Supplementary Figure 3.** Dairy food group (g/day of protein) contributors for total dairy protein, milk protein, and cheese protein intake. 34](#_Toc129632782)

[**Supplementary Figure 4.** Multivariable-adjusted hazard ratios (95% CI) for colorectal, breast and prostate cancer risk by **total** **grams** of dairy products, milk, and cheese. 35](#_Toc129632784)

[**Supplementary Figure 5.** Intake of total protein from per 2.5% energy increase by subgroups and risk of colorectal cancer, breast cancer, prostate cancer. 36](#_Toc129632785)

[**Supplementary Figure 6.** Intake of protein from total dairy products per 2.5% energy increase by subgroups and risk of colorectal cancer, breast cancer, prostate cancer. 37](#_Toc129632786)

[**Supplementary Figure 7.** Intake of milk protein per 2.5% energy increase by subgroups risk of colorectal cancer, breast cancer, prostate cancer. 38](#_Toc129632787)

[**Supplementary Figure 8.** Intake of cheese protein per 2.5% energy increase by subgroups risk of colorectal cancer, breast cancer, prostate cancer. 39](#_Toc129438942)

[**Supplementary Figure 9.** Intake of dietary calcium per 300 mg/day increase by subgroups risk of colorectal cancer, breast cancer, prostate cancer. 40](#_Toc129632788)

# Supplementary Methods

## Estimation of dietary intake

Participants needed to complete a minimum of two Oxford WebQ 24-hour dietary assessments (maximum of five) to be included in these analyses. Multiple 24-hour dietary assessments were used to reduce random measurement error in the estimates for intake of dietary exposures (i.e., protein, dairy protein and calcium) because of the day-to-day variation in dietary intake. Participants’ responses for each food and beverage were then converted to nutrient profiles based on the composition of the food or beverage. The estimation of intake of total protein, protein from all dairy sources, and calcium was determined by multiplying the serving size in grams (or milligrams for calcium) by the frequency in which it was reported in each of the 24-hour dietary assessment[1-3]. From participants’ responses, the average intake of total protein, protein from all dairy sources, protein from milk, protein from cheese, and total dietary calcium intake was estimated.

*Total protein intake*

Total protein intake was estimated from the protein contents of all foods reported in each 24-hour dietary assessment.

*Protein from all dairy product sources*

Protein from total dairy products was estimated by calculating the protein content of milk (whole, semi, skimmed), yogurt (low fat, and high fat), cheese (medium fat, and high fat), butter (low fat and normal), cream, and dairy desserts (ice cream and other dairy based desserts) based on what each participant reported in each 24-hour dietary assessment.

*Protein from milk*

Protein from milk was estimated based on the reported consumption of whole milk, semi-skimmed milk, or skimmed milk reported in each 24-hour dietary assessment. Participants were asked to report how many servings of milk they consumed the day before, and the amount of milk they added to their coffee, tea, or hot chocolate and if they added milk to their cereal.

*Protein from cheese*

Protein from cheese was estimated based on the reported consumption of a variety of cheeses asked in the 24-hour dietary assessment.

Grams of total protein and dairy protein sources were multiplied by 16.7 to obtain the kilojoule (kJ) of energy from the protein source. Once this was determined from all available 24-hour dietary assessments, total protein (in kJ) and protein from dairy (in kJ) was divided by the total amount of energy intake for the specific 24-hour dietary assessment for each participant to determine the percentage of energy from the dairy source for each individual 24-hour dietary assessment completed. From this, participants’ percentage of energy from total protein, total dairy protein, and protein from dairy sources (i..e, milk and cheese) were averaged across all available 24-hour dietary assessments to estimate their usual intake.

*Grams of dairy products, milk, and cheese*

Total grams of all dairy products, milk, and cheese were calculated based on the reported intake in each 24-hour dietary assessment. The serving size in grams was multiplied by the frequency and amount that was reported in each of the 24-hour dietary assessments to obtain the total grams of dairy products, milk, and cheese[1, 3].

*Calcium intake*

Dietary calcium intake, in milligram per day, was estimated from the calcium contents of all foods reported in each 24-hour dietary assessment.

## Other dietary variables

*Red and processed meat intake*

Total red and processed meat intake was estimated from participants’ responses to how often they consumed beef, lamb/mutton, pork, and processed meat from the options of: ‘never, ‘<once a week’, ‘once a week’, ‘2-4 times a week’, ‘5-6 times a week’ in the baseline questionnaire. This was estimated from the recruitment questionnaire rather than the 24-hour dietary assessment due to red and processed meat being consumed more episodically by these participants. The frequency of consumption of beef, pork, lamb, and processed meat were summed by assigning participants’ intakes as never as a 0, less than once a week a 0.5, once a week 1.0, 2-4 times a week a value of 3, and 5-6 times a week a value of 5.5. From this, intake of red and processed meat was categorised as <2.0 times per week, 2.0-2.9 times per week, 3.0-3.9 times per week, and ≥4.0 times per week and an unknown/missing category if participants reported ‘do not know’ or ‘prefer not to say’.

*Intake of fruit and vegetables*

Intake of fruit and vegetables were derived from each 24-hour dietary assessment. Intake of all fruits and vegetables in grams from: apples/pears, berries, citrus, dried fruit, other fruit, stewed fruit, allium vegetables, leafy greens, raw salad, root vegetables, tomatoes, other vegetables, and peas and corn were summed, and participants were categorised into quintiles of total grams of fruit and vegetables estimated from the average of all available 24-hour dietary assessments.

*Intake of fibre*

Fibre intake was estimated from each 24-hour dietary assessment using nutrient profiles from the reported foods consumed. The mean of fibre intake was determined across all available 24-hour dietary assessments and from this, participants were categorised into sex-specific quintiles of fibre intake.

## Covariates

*Region*

Region of participants were grouped based on the participants were based on the recruitment centre they attended. A total of 10 regions were used corresponding approximately to the areas covered by the assessment centre: London (assessment centres: St Bartholomew’s Hospital, Hounslow, Croydon) Wales (assessment centres: Swansea, Wrexham, Cardiff), North-West England (assessment centres: Stockport, Manchester, Liverpool, Bury), North-East England (assessment centres: Newcastle, Middlesbrough), Yorkshire (assessment centres: Leeds, Sheffield), West Midlands (assessment centres: Stoke, Birmingham) East Midlands (assessment centre: Nottingham), South-East England (assessment centres: Oxford, Reading), South-West England (assessment centre: Bristol), Scotland (assessment centres: Glasgow, Edinburgh).

*Height*

Participants were grouped into six sex-specific categories for height. For women categories were: <155, 155-159.9, 160-164.9, 165-169.9, 170-174.9, ≥175 cm. For men categories were: <165, 165-169.9, 170-174.9, 175-179.9, 180-184.9, ≥185 cm. From this, men and women were combined into one height variable with categories going from 1 to 6, and a missing category for participants where height measurement was missing.

*Body mass index (BMI)*

Both height and weight were measured by study personnel at the baseline visit and were used to determine participant’s BMI. BMI was calculated by taking the participants measured weight in kilograms and dividing it by the participants squared standing height in metres. Individuals with missing data were coded into a missing category. Participants were categorised as follows: <20, 20.0-22.49, 22.50-24.99, 25.00-27.49, 27.50-29.99, 30-32.49, 32.50-34.99, ≥35 kg/m^2^ and unknown/missing category if either weight or height was missing.

*Alcohol intake from the recruitment questionnaire*

Alcohol intake was estimated from the recruitment questionnaire rather than the 24-hour dietary assessments to better capture usual intake. A total of 18 questions on average alcoholic beverage consumption was asked at recruitment which may better capture usual alcohol intake over time as some individuals only consume alcohol episodically. Specifically, participants were asked on the recruitment questionnaire how often they drank alcohol with the possible responses being: “daily or almost daily”, “three or four times a week”, “once or twice a week”, “one to three times a month”, “special occasions only”, “never”, or “prefer not to answer”. Participants were also asked about their weekly and monthly intake of pints of beer, glasses of red wine, glasses of white wine/champagne, glasses of fortified wine, measures of spirits/liqueurs and glasses of other alcohol. A pint of beer was assumed to contain 20 grams of alcohol, and all other drinks contained 10 grams of alcohol, and then summed their total weekly and monthly consumption of alcohol accordingly. If the participant reported ‘do not know’ or ‘prefer not to answer’ to one of these questions on weekly or monthly consumption, they were coded as missing, except for ‘other alcohol’, in which case we assigned them 0 grams from other alcohol. We used participants reported weekly consumption of alcohol, if this was unknown (due to the participant reporting ‘do not know’ or ‘prefer not to answer’ for one or more of the relevant questions, except for ‘other alcohol’) we used monthly consumption, if available. To get an estimated daily total, we divided weekly consumption by 7 (or monthly consumption by 30.4375). Alcohol consumption was then categorised as sex-specific quintiles, none-drinkers or unknown. For participants who had unknown grams/day of alcohol but who reported consuming alcohol intake on ‘special occasions’, we assigned them to the lowest category.

*Physical activity*

Physical activity was determined from questions on the touchscreen questionnaire which asked about walking, moderate physical activity, and vigorous physical activity. Reported physical activity from these questions were used to estimate excess metabolic equivalent (MET)-hours/week of physical activity during work and leisure time. For each of the three physically activity categories (walking, moderate physical activity, and vigorous physical activity), participants were asked how many days in a typical week they did each of the activities for 10 minutes or more. For each category, participants who entered one or more days were then asked how many minutes they spent doing those activities on a typical day. For each activity category, the number of reported days was multiplied by the number of reported minutes on a typical day to generate duration of activity in minutes per week. Activity on a typical day of 1,260 min per week (equivalent to an average of 3 hours per day) were truncated at 1,260 min. Total MET values for each category from the International Physical Activity Questionnaire short form were: 3.3 for walking, 4.0 for moderate physical activity and 8.0 for vigorous physical activity[4]. Excess MET values were therefore 2.3 for walking, 3.0 for moderate physical activity and 7.0 for vigorous physical activity. Excess MET-hours per week were calculated by multiplying the excess MET value for each activity by the duration of activity in hours per week.

*Townsend deprivation index*

Townsend deprivation index was based on the preceding national census output areas. Each participant was assigned a score in correspondence to the output area in which their postcode was located. From this, participants were split into quintiles from most deprived to least deprived and to a missing category where postcode information was not provided.

*Smoking status*

Smoking was determined from questions from the recruitment questionnaire. Participants were asked “Do you smoke tobacco now?” and “in the past, how often have you smoked tobacco?” to determine their smoking status. Smokers were further divided based on how many cigarettes they said they smoked on average per day from the question ‘About how many cigarettes do you smoke on average each day?’.

*Ethnicity*

Ethnicity of participants was determined from questions in the touchscreen questionnaire ‘What is your ethnic group?’. Options included: White, mixed, Asian or Asian British, Black or Black British, Chinese, and other ethnic group. From this question, participants were grouped into four categories: White, mixed race or other, Asian or British Asian, and Black or Black British, or missing/unknown.

*Education*

For education, participants were asked ‘Which of the following qualification do you have?’ being able to select more than one. Possible answers were: College or University degree; A levels/AS levels or equivalent; O levels/GCSEs or equivalent; CSEs or equivalent; NVQ or HND or HNC or equivalent; Other professional qualifications example: nursing, teaching; None of the above; Prefer not to answer. We grouped participants into the following categories, based on their highest reported level of education: (College or University degree, vocational qualifications (other professional qualifications/NVQ or HND or HNC), optional national exams at ages 17 to 18 years (A levels/AS levels), national exams at age 16 years (O levels/GCSEs/CSEs), none of the above, prefer not to answer, and missing (unknown/missing)).

*Employment status*

Employment status at recruitment was assessed by asking participants “Which of the

following describes your current situation?” in which they selected answers which were applicable to them including ‘In paid employment or self-employed’, ‘retired’, ‘looking after home and/or family’, ‘unable to work because of sickness or disability’, ‘unemployed’, ‘doing unpaid or voluntary work’, ‘full or part time student’, ‘none of the above’ or ‘prefer not to answer’. Participants were defined as being “in paid employment” if they responded they were in paid employment or self-employed, “retired” if they responded they were retired, and “not in paid employment” if they reported being unemployed, inability to work, being a student, or having caring responsibilities for their family. Finally, an unknown/missing category consisted of participants who did not respond, said they prefer not to answer, or answered none of the above options in the question.

*Diabetes status*

Participants diabetes status was determined using multiple questions from recruitment. First, from the question ‘Has a doctor ever told you that you have diabetes?’ participants were classified as ‘yes’, ‘no’ or ‘unknown’ based on their response. As well, participants who reported using metformin or insulin at recruitment were considered diabetic and included in the ‘yes’ category. Finally, if a participant had a measured glycated hemoglobin (HbA1c) of ≥ 48 mmol/mol at recruitment, they were defined as being diabetic and included in the ‘yes’ category.

*Non-steroid anti-inflammatory drug use*

Non-steroid anti-inflammatory drug (NSAID) use was determined based on the medications reported at recruitment. Participants were categorised into three groups “Non-users”, “irregular NSAID users”, and “regular users of aspirin or ibuprofen”. Participants were categorised as “regular users of aspirin or ibuprofen” if they responded to taking ibuprofen or aspirin regularly in the touchscreen questionnaire completed at recruitment. Participants were categorised into irregular users if they responded to taking any classification of NSAID at recruitment. Non-users were categorised if they did not report any use of NSAIDs.

*Marital status*

Living with a partner was derived from a question asked at recruitment in which participants answered if they lived with anyone else in their household. If the participants reported to be living with a husband, wife or partner in their household they were classified as ‘living with a partner’, if the participant reported to be living with any other person or alone they were categorised as ‘not living with a partner’.

**Women specific covariates**

For menopausal hormone therapy and menopausal status, all men were put into a separate category for these women-specific covariates as they were controlled for in the colorectal cancer models including both sexes.

*Menopausal hormone therapy*

Use of menopausal hormone therapy (MHT) was categorised as ‘current user’, ‘former user’ and ‘never user’ or ‘unknown/missing’ based on the questions asked about MHT use in the touchscreen questionnaire. Women were asked ‘Have you ever used hormone replacement therapy (HRT)?’ and if they answer yes: ‘How old were you when you last used HRT?’. Women were asked to enter their age when they last used HRT or could choose between ‘Still taking HRT’, or they could select ‘prefer not to answer’ or ‘do not know’. From this, women were categorised into respective groups.

*Menopausal status at recruitment*

Menopausal status was determined by multiple questions asked in the baseline questionnaire Women were defined as being pre-menopausal if they:

- Answered ‘no’ to the question regarding having gone through menopause, or
- Reported they were ‘not sure’ or did not respond to if they had gone through menopause and:
  - Were <50 years of age, did not have a bilateral oophorectomy/hysterectomy, and reported they were not using menopausal hormone therapy.
  - Were <50 years of age, reported they were menstruating today, and did not have a bilateral oophorectomy/hysterectomy.

Women were defined as post-menopausal if they:

- Answered ‘yes’ to having gone through menopause
- Answered ‘not sure’ or did not answer if they had gone through menopause and:
  - Were ≥55 years of age, or
  - Had a bilateral oophorectomy

Women were defined as their menopausal status being unknown if:

- Answered ‘no’ to having gone through menopause and:
  - Did not answer no to using HRT, or
  - Did not answer no to having a bilateral oophorectomy, or
  - Did not answer no to having a hysterectomy, or
  - Were 50-54.9 years of age.

When assessing heterogeneity by menopause status, participants follow-up time were split at age 55 to account for change in menopausal status during follow-up, (approximately 98% of women having undergone menopause by this age). Thus, if a woman’s menopausal status was defined at recruitment as premenopausal or unknown and over follow-up they turned 55 years old, they were added to the postmenopausal risk set at this time. For example, if a 49-year-old woman defined as premenopausal at recruitment, contributed 10 years by the end of follow-up, 6 years they would be defined as premenopausal and at age 55 they would switch to being postmenopausal and contribute 4 years of follow-up being categorised as postmenopausal. If women were defined as postmenopausal at recruitment, they remained in the postmenopausal category throughout their follow-up time.

*Parity and age at first birth*

Parity was defined by the recruitment question of “how many children have you given birth to?”.

Women who said they had a given birth were asked how old they were in years when they gave birth to their first child. Based on these responses, women were categorised into groups of: 0 children (Nulliparous), 1-2 children <25 years of age, or ≥3 children <25 years of age, 1-2 children 25-29.9 years of age, or ≥3 children 25-29.9 years of age, 1-2 children ≥30years of age, or ≥3 children ≥30 years of age or unknown if the participants responded, ‘do not know’ or ‘prefer not to answer’.

*Age at menarche*

Women were categorised based on the recruitment question “How old were you when your period started”. From this, women were categorised into age groups as: ≤12 years old, 13 years old, ≥14 years old. If a participant responded ‘prefer not to answer’ or ‘do not know’ they were categorised into an unknown group.

Breast cancer analyses:

*Interaction between BMI and menopause status*

To account for the heterogeneity between BMI and menopause on breast cancer risk, women were categorised into 6 categories. Women who were categorised as premenopausal:<25, 25-29.9, ≥30 kg/m^2^ and postmenopausal: <25, 25-29.9, ≥30 kg/m^2^; due to the limited number of premenopausal women, only three categories were made. If a woman had an unknown menopause status and/or BMI they were categorised into an unknown category. This variable replaced menopause in the model for breast cancer analyses

**References**

1. Perez-Cornago A, Pollard Z, Young H, van Uden M, Andrews C, Piernas C, et al. Description of the updated nutrition calculation of the Oxford WebQ questionnaire and comparison with the previous version among 207,144 participants in UK Biobank. Eur J Nutr. 2021;60: 4019-30. <https://doi.org/10.1007/s00394-021-02558-4>

2. Piernas C, Perez-Cornago A, Gao M, Young H, Pollard Z, Mulligan A, et al. Describing a new food group classification system for UK biobank: analysis of food groups and sources of macro- and micronutrients in 208,200 participants. European Journal of Nutrition. 2021;60: 2879-90. <https://doi.org/10.1007/s00394-021-02535-x>

3. Swan G, Dodhia S, Farron-Wilson M, Powell N, Bush M. Food composition data and public health. Nutrition Bulletin. 2015;40: 223-6. <https://doi.org/10.1111/nbu.12156>

4. IPAQ Research Committee. Guidelines for data processing and analysis of the International Physical Activity Questionnaire (IPAQ)— Short and Long Forms. 2005.

# Supplementary Tables

| **Supplementary Table 1.** Baseline characteristics of **women** by lowest and highest quartile of percentage of energy intake from protein from all dairy products, milk, and cheese and total dietary calcium intake | | | | | | | | | | | |
| --- | --- | --- | --- | --- | --- | --- | --- | --- | --- | --- | --- |
|  | **Protein from dairy products** | |  | **Protein from milk** | |  | **Protein from cheese** | |  | **Total dietary calcium** | |
|  | **Q1** | **Q4** |  | **Q1** | **Q4** |  | **Q1** | **Q4** |  | **Q1** | **Q4** |
| Number of participants | 15,735 | 15,734 |  | 15,735 | 15,734 |  | 16,162 | 15,734 |  | 15,735 | 15,734 |
| Age at recruitment - years | 54.3 (7.8) | 56.2 (7.5) |  | 54.7 (7.8) | 56.2 (7.5) |  | 55.3 (7.7) | 55.3 (7.7) |  | 54.9 (7.7) | 55.9 (7.7) |
| Body mass index - kg/m^2^ | 26.3 (5.1) | 26.2 (4.8) |  | 26.2 (5.1) | 26.2 (4.7) |  | 26.6 (5.0) | 26.0 (4.9) |  | 26.4 (4.9) | 26.1 (4.9) |
| Height - centimetres | 163.4 (6.2) | 163.4 (6.1) |  | 163.6 (6.3) | 163.2 (6.1) |  | 163.0 (6.2) | 163.8 (6.1) |  | 162.9 (6.2) | 164.1 (6.2) |
| Physical Activity - High, N (%) | 2537 (16.1%) | 2588 (16.4%) |  | 2721 (17.3%) | 2502 (15.9%) |  | 2634 (16.3%) | 2525 (16.0%) |  | 2392 (15.2%) | 2781 (17.7%) |
| Townsend deprivation index, N (%) |  |  |  |  |  |  |  |  |  |  |  |
| Q1 - Most affluent | 3071 (19.5%) | 3522 (22.4%) |  | 2926 (18.6%) | 3735 (23.7%) |  | 3408 (21.1%) | 3229 (20.5%) |  | 3058 (19.4%) | 3425 (21.8%) |
| Q5 - Most deprived | 3036 (19.3%) | 2355 (15.0%) |  | 3130 (19.9%) | 2179 (13.8%) |  | 2726 (16.9%) | 2711 (17.2%) |  | 3016 (19.2%) | 2318 (14.7%) |
| Paid employment, N (%) | 10334 (65.7%) | 9216 (58.6%) |  | 10215 (64.9%) | 9060 (57.6%) |  | 9929 (61.4%) | 9906 (63.0%) |  | 10224 (65.0%) | 9324 (59.3%) |
| University/college degree N (%) | 11037 (70.1%) | 11135 (70.8%) |  | 11502 (73.1%) | 10784 (68.5%) |  | 10539 (65.2%) | 11732 (74.6%) |  | 10690 (67.9%) | 11560 (73.5%) |
| White ethnicity, N (%) | 14785 (94.0%) | 15416 (98.0%) |  | 14789 (94.0%) | 15479 (98.4%) |  | 15319 (94.8%) | 15327 (97.4%) |  | 14834 (94.3%) | 15359 (97.6%) |
| Never smoker, N (%) | 9267 (58.9%) | 9907 (63.0%) |  | 9267 (58.9%) | 10128 (64.4%) |  | 10001 (61.9%) | 9438 (60.0%) |  | 9108 (57.9%) | 10132 (64.4%) |
| Diabetes diagnosed – Yes, N (%) | 420 (2.7%) | 410 (2.6%) |  | 368 (2.3%) | 449 (2.9%) |  | 489 (3.0%) | 381 (2.4%) |  | 427 (2.7%) | 427 (2.7%) |
| Living with a partner, N (%) | 10985 (69.8%) | 11034 (70.1%) |  | 10736 (68.2%) | 11434 (72.7%) |  | 11325 (70.1%) | 10874 (69.1%) |  | 11019 (70.0%) | 11059 (70.3%) |
| **Diet variables** |  |  |  |  |  |  |  |  |  |  |  |
| Alcohol intake - g/day | 12.5 (11.8) | 9.3 (9.0) |  | 12.2 (11.7) | 8.9 (8.6) |  | 10.4 (10.4) | 11.2 (10.6) |  | 12.4 (11.7) | 9.6 (9.2) |
| Red and processed meat intake - times/week | 3.1 (2.1) | 2.8 (1.9) |  | 2.8 (2.1) | 3.0 (1.9) |  | 3.1 (2.0) | 2.8 (2.0) |  | 3.1 (2.0) | 2.9 (2.0) |
| Vegetable and fruit intake - g/day | 409.5 (239.9) | 418.3 (229.1) |  | 441.9 (253.8) | 384.5 (207.3) |  | 407.7 (242.0) | 414.2 (227.3) |  | 353.5 (203.9) | 478.7 (257.2) |
| Total dairy product intake - g/day | 166.0 (94.9) | 469.0 (167.6) |  | 177.1 (127.5) | 452.4 (153.3) |  | 300.5 (171.8) | 331.7 (163.8) |  | 181.6 (97.6) | 470.7 (175.0) |
| Total milk intake - g/day | 95.1 (81.6) | 271.9 (125.7) |  | 46.4 (44.7) | 324.1 (94.9) |  | 186.7 (121.5) | 185.2 (118.4) |  | 110.4 (83.6) | 270.4 (132.0) |
| Total cheese intake - g/day | 7.2 (9.2) | 28.7 (21.3) |  | 18.4 (18.7) | 15.0 (15.5) |  | 0.0 (0.0) | 39.0 (16.6) |  | 9.1 (11.4) | 26.9 (21.1) |
| Total protein intake - % of energy/day | 15.6 (3.2) | 17.2 (3.2) |  | 15.7 (3.3) | 17.3 (3.1) |  | 16.7 (3.4) | 16.3 (3.2) |  | 16.4 (3.6) | 16.2 (2.8) |
| Dairy product protein - % of energy/day | 1.4 (0.6) | 5.0 (1.0) |  | 2.0 (1.2) | 4.3 (1.3) |  | 2.3 (1.3) | 4.2 (1.3) |  | 2.1 (1.1) | 4.1 (1.4) |
| Milk protein - % of energy/day | 0.7 (0.6) | 2.1 (1.0) |  | 0.3 (0.3) | 2.6 (0.6) |  | 1.5 (1.0) | 1.4 (0.9) |  | 1.0 (0.8) | 1.8 (1.0) |
| Cheese protein - % of energy/day | 0.3 (0.4) | 1.6 (1.1) |  | 0.9 (1.0) | 0.8 (0.9) |  | 0.0 (0.0) | 2.1 (0.8) |  | 0.5 (0.7) | 1.3 (1.0) |
| Total carbohydrate intake - % of energy/day | 48.8 (7.7) | 50.5 (7.4) |  | 48.6 (8.2) | 51.3 (6.8) |  | 51.0 (7.6) | 48.0 (7.6) |  | 48.1 (8.5) | 51.0 (6.5) |
| Total fat intake - % of energy/day | 32.2 (5.9) | 31.3 (6.0) |  | 32.7 (6.2) | 30.4 (5.5) |  | 30.4 (5.9) | 33.4 (5.9) |  | 31.6 (6.3) | 32.1 (5.5) |
| Total fibre intake - g/day | 17.8 (5.8) | 17.1 (5.3) |  | 18.3 (6.0) | 16.6 (5.0) |  | 17.1 (5.7) | 17.5 (5.4) |  | 14.5 (4.6) | 20.7 (5.7) |
| Total dietary calcium intake – mg/day | 748 (212) | 1136 (264) |  | 815 (254) | 1043 (266) |  | 823 (247) | 1060 (277) |  | 627 (101) | 1298 (176) |
| Total energy intake - kJ/day | 8139 (1684) | 7640 (1585) |  | 8118 (1703) | 7412 (1475) |  | 7632 (1636) | 8015 (1665) |  | 6846 (1327) | 9219 (1493) |
| Values are mean (SD) unless otherwise indicated. | | | | | | | | | | | |
| Abbreviations: g/day, grams per day; kg/m^2^, kilograms per metre squared; kJ/day, kilojoules per day; mg/day, milligrams per day; N, Number of participants; Q, quantile; y, years. | | | | | | | | | | | |

| **Supplementary Table 2.** Baseline characteristics **of men** by lowest and highest quartile of percentage of energy intake from protein from all dairy products, milk, and cheese and total dietary calcium intake. | | | | | | | | | | | |
| --- | --- | --- | --- | --- | --- | --- | --- | --- | --- | --- | --- |
|  | **Protein from dairy products** | |  | **Protein from milk** | |  | **Protein from cheese** | |  | **Total dietary calcium** | |
|  | **Q1** | **Q4** |  | **Q1** | **Q4** |  | **Q1** | **Q4** |  | **Q1** | **Q4** |
| Number of participants | 12,820 | 12,819 |  | 12,820 | 12,819 |  | 14,796 | 12,819 |  | 12820 | 12819 |
| Age at recruitment - years | 55.6 (8.1) | 57.3 (7.7) |  | 55.7 (8.1) | 57.2 (7.7) |  | 56.2 (8.0) | 56.5 (7.9) |  | 56.3 (7.9) | 56.4 (8.0) |
| Body mass index - kg/m^2^ | 27.4 (4.2) | 27.2 (4.0) |  | 27.4 (4.2) | 27.2 (3.9) |  | 27.4 (4.1) | 27.2 (4.1) |  | 27.6 (4.2) | 27.0 (4.1) |
| Height - centimetres | 176.4 (6.6) | 176.7 (6.7) |  | 176.7 (6.7) | 176.4 (6.7) |  | 176.1 (6.6) | 177.0 (6.7) |  | 175.7 (6.7) | 177.5 (6.7) |
| Physical Activity - High, N (%) | 2364 (18.4%) | 2330 (18.2%) |  | 2405 (18.8%) | 2303 (18.0%) |  | 2741 (18.5%) | 2291 (17.9%) |  | 2061 (16.1%) | 2778 (21.7%) |
| Townsend deprivation index, N (%) |  |  |  |  |  |  |  |  |  |  |  |
| Q1 - Most affluent | 2609 (20.4%) | 3033 (23.7%) |  | 2539 (19.8%) | 3108 (24.2%) |  | 3308 (22.4%) | 2838 (22.1%) |  | 2745 (21.4%) | 3004 (23.4%) |
| Q5 - Most deprived | 2397 (18.7%) | 1884 (14.7%) |  | 2530 (19.7%) | 1753 (13.7%) |  | 2516 (17.0%) | 2150 (16.8%) |  | 2318 (18.1%) | 1893 (14.8%) |
| Paid employment, N (%) | 8504 (66.3%) | 7527 (58.7%) |  | 8433 (65.8%) | 7629 (59.5%) |  | 9416 (63.6%) | 8112 (63.3%) |  | 8256 (64.4%) | 7916 (61.8%) |
| University/college degree, N (%) | 9590 (74.8%) | 9964 (77.7%) |  | 9927 (77.4%) | 9729 (75.9%) |  | 10749 (72.6%) | 10248 (79.9%) |  | 9639 (75.2%) | 9979 (77.8%) |
| White ethnicity, N (%) | 12141 (94.7%) | 12507 (97.6%) |  | 12121 (94.5%) | 12528 (97.7%) |  | 14012 (94.7%) | 12504 (97.5%) |  | 12120 (94.5%) | 12476 (97.3%) |
| Never smoker, N (%) | 6315 (49.3%) | 7018 (54.7%) |  | 6241 (48.7%) | 7187 (56.1%) |  | 8009 (54.1%) | 6535 (51.0%) |  | 6383 (49.8%) | 7098 (55.4%) |
| Diabetes diagnosed – Yes, N (%) | 676 (5.3%) | 706 (5.5%) |  | 615 (4.8%) | 750 (5.9%) |  | 890 (6.0%) | 700 (5.5%) |  | 748 (5.8%) | 577 (4.5%) |
| Living with a partner, N (%) | 9783 (76.3%) | 10107 (78.8%) |  | 9798 (76.4%) | 10178 (79.4%) |  | 11322 (76.5%) | 10093 (78.7%) |  | 9890 (77.1%) | 9964 (77.7%) |
| **Diet variables** |  |  |  |  |  |  |  |  |  |  |  |
| Alcohol intake - g/day | 27.3 (23.7) | 17.6 (16.6) |  | 26.6 (24.8) | 17.3 (15.8) |  | 22.2 (21.8) | 21.8 (19.7) |  | 25.2 (22.2) | 19.4 (17.7) |
| Red and processed meat intake – times/week | 4.2 (2.4) | 3.8 (2.2) |  | 3.9 (2.4) | 3.9 (2.1) |  | 4.1 (2.3) | 3.8 (2.3) |  | 4.0 (2.3) | 3.9 (2.3) |
| Vegetable and fruit intake - g/day | 342.6 (226.8) | 351.1 (209.1) |  | 370.5 (239.6) | 331.7 (196.7) |  | 341.4 (222.1) | 353.3 (212.0) |  | 296.9 (190.9) | 403.2 (241.1) |
| Total dairy product intake - g/day | 171.0 (93.2) | 476.4 (183.6) |  | 180.3 (125.4) | 463.1 (169.0) |  | 303.0 (173.2) | 339.3 (168.6) |  | 185.2 (96.6) | 477.0 (189.9) |
| Total milk intake - g/day | 112.3 (82.9) | 293.7 (143.1) |  | 64.6 (51.7) | 345.8 (116.5) |  | 205.9 (128.6) | 201.7 (123.7) |  | 124.1 (85.2) | 291.7 (147.2) |
| Total cheese intake - g/day | 6.5 (9.2) | 31.6 (23.4) |  | 19.9 (20.9) | 15.2 (16.6) |  | 0.0 (0.00) | 42.3 (18.1) |  | 8.4 (11.0) | 29.7 (23.4) |
| Total protein intake - % of energy/day | 15.0 (3.1) | 16.4 (2.9) |  | 15.0 (3.1) | 16.5 (2.9) |  | 15.8 (3.2) | 15.7 (2.9) |  | 1.0 (0.7) | 1.6 (0.9) |
| Dairy product protein - % of energy/day | 1.2 (0.5) | 4.5 (0.9) |  | 1.8 (1.1) | 3.8 (1.2) |  | 2.0 (1.1) | 3.8 (1.2) |  | 1.8 (1.0) | 3.6 (1.3) |
| Milk protein - % of energy/day | 0.7 (0.5) | 2.0 (1.0) |  | 0.4 (0.3) | 2.4 (0.7) |  | 1.4 (0.9) | 1.3 (0.8) |  | 1.0 (0.7) | 1.6 (0.9) |
| Cheese protein - % of energy/day | 0.3 (0.4) | 1.5 (1.1) |  | 0.9 (0.9) | 0.8 (0.8) |  | 0.0 (0.00) | 2.0 (0.7) |  | 0.5 (0.6) | 1.2 (1.0) |
| Total carbohydrate intake - % of energy/day | 47.9 (7.9) | 49.9 (7.3) |  | 47.4 (8.2) | 51.0 (6.9) |  | 50.5 (7.7) | 47.3 (7.3) |  | 47.6 (8.6) | 50.1 (6.6) |
| Total fat intake - % of energy/day | 30.7 (6.0) | 31.4 (5.8) |  | 31.6 (6.2) | 30.2 (5.5) |  | 29.6 (5.9) | 32.9 (5.7) |  | 30.2 (6.3) | 32.1 (5.4) |
| Total fibre intake - g/day | 18.1 (6.3) | 17.9 (5.7) |  | 18.6 (6.6) | 17.4 (5.5) |  | 17.5 (6.1) | 18.4 (6.0) |  | 14.7 (4.8) | 21.8 (6.2) |
| Total dietary calcium intake - mg/day | 812 (239) | 1232 (303) |  | 891 (289) | 1115 (305) |  | 885 (270) | 1166 (308) |  | 669 (110) | 1423 (209) |
| Total energy intake - kJ/day | 9493 (2101) | 8916 (1960) |  | 9574. (2148) | 8534 (1823) |  | 8870 (2031) | 9409 (2042) |  | 7795 (1585) | 10938 (1862) |
| Values are mean (SD) unless otherwise indicated. | | | | | | | | | | | |
| Abbreviations: g/day, grams per day; kg/m^2^, kilojoules per metre squared; kJ/day, kilojoules per day; mg/day, milligrams per day; N, Number of participants; Q, quantile; y, years. | | | | | | | | | | | |

| **Supplementary Table 3.** Dairy protein intake in grams and percentage of energy per day by quartiles of intake in all participants and separately by males and females. | | | | | | | | | | | | | | | |
| --- | --- | --- | --- | --- | --- | --- | --- | --- | --- | --- | --- | --- | --- | --- | --- |
|  | | **All** | | | |  | **Male** | | | |  | **Female** | | | |
|  | | **Q1** | **Q2** | **Q3** | **Q4** |  | **Q1** | **Q2** | **Q3** | **Q4** |  | **Q1** | **Q2** | **Q3** | **Q4** |
| **Total protein** | | |  |  |  |  |  |  |  |  |  |  |  |  |  |
|  | N | 28,555 | 28,554 | 28,554 | 28,554 |  | 12,820 | 12,819 | 12,820 | 12,819 |  | 15,735 | 15,735 | 15,735 | 15,734 |
|  | g/d | 68.6 (16.5) | 79.0 (16.5) | 84.2 (17.5) | 90.0 (20.4) |  | 72.5 (16.8) | 83.4 (16.7) | 89.2 (18.2) | 96.3 (22.3) |  | 64.1 (14.2) | 75.0 (14.3) | 80.2 (15.1) | 86.5 (17.7) |
|  | % of total energy intake/d | 12.42 (1.2) | 14.8 (0.5) | 16.64 (0.6) | 19.98 (2.2) |  | 12.21 (1.2) | 14.5 (0.5) | 16.22 (0.5) | 19.39 (2.2) |  | 12.62 (1.2) | 15.1 (0.5) | 17.0 (0.6) | 20.4 (2.3) |
| **Protein from dairy products** | | |  |  |  |  |  |  |  |  |  |  |  |  |  |
|  | N | 28,555 | 28,554 | 28,554 | 28,554 |  | 12,820 | 12,819 | 12,820 | 12,819 |  | 15,735 | 15,735 | 15,735 | 15,734 |
|  | g/d | 7.0 (3.2) | 12.6 (3.1) | 16.4 (3.8) | 22.4 (6.2) |  | 6.9 (3.1) | 12.5 (3.0) | 16.6 (3.9) | 23.2 (6.5) |  | 6.9 (3.2) | 12.5 (2.8) | 16.2 (3.5) | 22.1 (5.8) |
|  | % of total energy intake/d | 1.31 (0.5) | 2.4 (0.2) | 3.25 (0.3) | 4.75 (1.0) |  | 1.22 (0.5) | 2.2 (0.2) | 3.02 (0.2) | 4.46 (0.9) |  | 1.41 (0.6) | 2.6 (0.2) | 3.4 (0.3) | 5.0 (1.0) |
| **Protein from milk** | | |  |  |  |  |  |  |  |  |  |  |  |  |  |
|  | N | 28,555 | 28,554 | 28,554 | 28,554 |  | 12,820 | 12,819 | 12,820 | 12,819 |  | 15,735 | 15,735 | 15,735 | 15,734 |
|  | g/d | 1.9 (1.7) | 5.8 (1.5) | 8.2 (1.8) | 11.4 (3.6) |  | 2.2 (1.8) | 6.1 (1.5) | 8.4 (1.8) | 11.8 (3.9) |  | 1.6 (1.5) | 5.5 (1.4) | 8.0 (1.7) | 11.1 (3.2) |
|  | % of total energy intake/d | 0.35 (0.3) | 1.1 (0.2) | 1.62 (0.2) | 2.52 (0.6) |  | 0.38 (0.3) | 1.1 (0.1) | 1.54 (0.1) | 2.40 (0.6) |  | 0.32 (0.3) | 1.1 (0.2) | 1.7 (0.2) | 2.6 (0.6) |
| **Protein from cheese** | | |  |  |  |  |  |  |  |  |  |  |  |  |  |
|  | N | 30,958 | 26,151 | 28,554 | 28,554 |  | 14,796 | 10,843 | 12,820 | 12,819 |  | 16,162 | 15,308 | 15,735 | 15,734 |
|  | g/d | 0.0 (0.0) | 2.4 (1.0) | 5.1 (1.6) | 10.3 (4.3) |  | 0.0 (0.0) | 2.5 (1.0) | 5.3 (1.6) | 11.0 (4.5) |  | 0.0 (0.0) | 2.3 (1.0) | 4.9 (1.4) | 9.8 (3.9) |
|  | % of total energy intake/d | 0.0 (0.0) | 0.4 (0.2) | 0.97 (0.2) | 2.05 (0.7) |  | 0.0 (0.0) | 0.4 (0.1) | 0.93 (0.2) | 2.00 (0.7) |  | 0.0 (0.0) | 0.4 (0.2) | 1.0 (0.2) | 2.1 (0.8) |
| **Total dietary calcium** | |  |  |  |  |  |  |  |  |  |  |  |  |  |  |
|  | N | 28,555 | 28,554 | 28,554 | 28,554 |  | 12,820 | 12,819 | 12,820 | 12,819 |  | 15,735 | 15,735 | 15,735 | 15,734 |
|  | mg/d | 643.9 (104.7) | 867.3 (49.8) | 1,044.1 (55.8) | 1,359.0 (196.1) |  | 669.3 (110.0) | 903.3 (52.1) | 1,090.3 (59.3) | 1,423.4 (207.8) |  | 626.7 (100.9) | 840.8 (47.7) | 1,008.3 (51.5) | 1,298.2 (175.9) |
| Values represent mean (SD). Grams per day represent the average grams of protein consumed from all sources, dairy source within each quantile. % of total energy represents the average percentage of energy consumed from dairy protein source within each quantile. Milligrams per day represent the milligrams of calcium consumed from all foods.  Abbreviations: g/d, grams per day; mg/d milligrams per day; Q, quantile; SD, standard deviation. | | | | | | | | | | | | | | | |

| **Supplementary Table 4.** Hazard ratios and 95% confidence intervals for minimally adjusted and sequential adjustment models for total protein, protein from all dairy products, milk, and cheese, and total dietary calcium intake and risk of **colorectal cancer.** | | | | | | | | |
| --- | --- | --- | --- | --- | --- | --- | --- | --- |
| **Colorectal cancer** | Intakes of total protein, protein from all dairy products, milk, cheese, and dietary calcium | | | | | |  |  |
|  | Q1 | Q2 | Q3 | Q4 |  | Per 2.5% energy increase | χ^2^ | *P-trend* |
| **Total protein** |  |  |  |  |  |  |  |  |
| Minimally-adjusted model | 1 (ref) | 1.12 (0.96 - 1.31) | 0.98 (0.84 - 1.16) | 0.95 (0.81 - 1.13) |  | 0.97 (0.92 - 1.02) | 1.80 | 0.18 |
| Multivariable-adjusted model 1 | 1 (ref) | 1.13 (0.97 - 1.32) | 1.01 (0.85 - 1.18) | 1.01 (0.85 - 1.20) |  | 0.99 (0.94 - 1.04) | 0.29 | 0.59 |
| Multivariable-adjusted model 2  (+ smoking and alcohol intake) | 1 (ref) | 1.15 (0.98 - 1.34) | 1.03 (0.88 - 1.22) | 1.04 (0.87 - 1.24) |  | 0.99 (0.94 - 1.05) | 0.05 | 0.83 |
| Multivariable-adjusted model 3 (+ BMI) | 1 (ref) | 1.14 (0.97 - 1.33) | 1.02 (0.86 - 1.20) | 1.01 (0.85 - 1.20) |  | 0.98 (0.93 - 1.04) | 0.37 | 0.54 |
| Multivariable-adjusted model 4  (+ red and processed meat; fully adjusted) | 1 (ref) | 1.13 (0.96 - 1.32) | 1.00 (0.85 - 1.18) | 0.99 (0.83 - 1.18) |  | 0.98 (0.93 - 1.03) | 0.79 | 0.37 |
| **Protein from dairy products** |  |  |  |  |  |  |  |  |
| Minimally-adjusted model | 1 (ref) | 0.90 (0.77 - 1.05) | 0.85 (0.73 - 1.00) | **0.75 (0.63 - 0.88)** |  | **0.79 (0.71 - 0.88)** | 17.64 | <0.001 |
| Multivariable-adjusted model 1 | 1 (ref) | 0.89 (0.77 - 1.04) | 0.85 (0.73 - 1.00) | **0.76 (0.64 - 0.90)** |  | **0.80 (0.72 - 0.90)** | 15.62 | <0.001 |
| Multivariable-adjusted model 2  (+ smoking and alcohol intake) | 1 (ref) | 0.91 (0.78 - 1.07) | 0.88 (0.75 - 1.03) | **0.79 (0.67 - 0.94)** |  | **0.82 (0.74 - 0.92)** | 11.74 | 0.001 |
| Multivariable-adjusted model 3 (+ BMI) | 1 (ref) | 0.91 (0.78 - 1.07) | 0.88 (0.75 - 1.03) | **0.79 (0.67 - 0.94)** |  | **0.82 (0.74 - 0.92)** | 11.84 | 0.001 |
| Multivariable-adjusted model 4  (+ red and processed meat; fully adjusted) | 1 (ref) | 0.91 (0.78 - 1.06) | 0.88 (0.75 - 1.03) | **0.80 (0.67 - 0.94)** |  | **0.82 (0.74 - 0.92)** | 11.61 | 0.001 |
| **Protein from milk** |  |  |  |  |  |  |  |  |
| Minimally-adjusted model | 1 (ref) | 0.88 (0.76 - 1.03) | **0.79 (0.67 - 0.92)** | **0.75 (0.64 - 0.89)** |  | **0.72 (0.61 - 0.86)** | 13.60 | <0.001 |
| Multivariable-adjusted model 1 | 1 (ref) | 0.87 (0.74 - 1.01) | **0.78 (0.67 - 0.92)** | **0.77 (0.65 - 0.91)** |  | **0.74 (0.62 - 0.88)** | 11.66 | 0.001 |
| Multivariable-adjusted model 2  (+ smoking and alcohol intake) | 1 (ref) | 0.88 (0.75 - 1.02) | **0.80 (0.69 - 0.94)** | **0.80 (0.68 - 0.95)** |  | **0.77 (0.64 - 0.92)** | 8.27 | 0.004 |
| Multivariable-adjusted model 3 (+ BMI) | 1 (ref) | 0.88 (0.75 - 1.02) | **0.81 (0.69 - 0.94)** | **0.80 (0.68 - 0.95)** |  | **0.77 (0.64 - 0.92)** | 8.23 | 0.004 |
| Multivariable-adjusted model 4  (+ red and processed meat; fully adjusted) | 1 (ref) | 0.87 (0.74 - 1.01) | **0.80 (0.68 - 0.93)** | **0.79 (0.67 - 0.94)** |  | **0.76 (0.63 - 0.91)** | 9.01 | 0.003 |
| **Protein from cheese** |  |  |  |  |  |  |  |  |
| Minimally-adjusted model | 1 (ref) | 0.94 (0.80 - 1.10) | 0.98 (0.84 - 1.15) | 0.91 (0.78 - 1.07) |  | 0.91 (0.77 - 1.08) | 1.08 | 0.30 |
| Multivariable-adjusted model 1 | 1 (ref) | 0.91 (0.77 - 1.06) | 0.95 (0.81 - 1.11) | 0.89 (0.75 - 1.04) |  | 0.90 (0.76 - 1.07) | 1.44 | 0.23 |
| Multivariable-adjusted model 2  (+ smoking and alcohol intake) | 1 (ref) | 0.91 (0.77 - 1.06) | 0.95 (0.81 - 1.11) | 0.88 (0.75 - 1.04) |  | 0.89 (0.75 - 1.06) | 1.64 | 0.20 |
| Multivariable-adjusted model 3 (+ BMI) | 1 (ref) | 0.91 (0.77 - 1.07) | 0.95 (0.81 - 1.11) | 0.89 (0.76 - 1.04) |  | 0.90 (0.76 - 1.07) | 1.52 | 0.22 |
| Multivariable-adjusted model 4  (+ red and processed meat; fully adjusted) | 1 (ref) | 0.91 (0.77 - 1.07) | 0.95 (0.82 - 1.12) | 0.89 (0.76 - 1.05) |  | 0.91 (0.76 - 1.08) | 1.25 | 0.26 |
|  |  |  |  |  |  |  |  |  |
| **Total dietary calcium** | Q1 | Q2 | Q3 | Q4 |  | Per 300 mg/day increase | χ^2^ | *P-trend* |
| Minimally-adjusted model | 1 (ref) | 0.97 (0.82 - 1.13) | 0.96 (0.82 - 1.13) | **0.84 (0.71 - 0.99)** |  | 0.95 (0.90 - 1.01) | 2.26 | 0.133 |
| Multivariable-adjusted model 1 | 1 (ref) | 0.87 (0.73 - 1.02) | **0.81 (0.68 - 0.96)** | **0.65 (0.54 - 0.79)** |  | **0.86 (0.80 - 0.93)** | 15.26 | <0.001 |
| Multivariable-adjusted model 2  (+ smoking and alcohol intake) | 1 (ref) | 0.89 (0.75 - 1.05) | 0.84 (0.71 - 1.01) | **0.69 (0.57 - 0.84)** |  | **0.89 (0.82 - 0.96)** | 9.73 | 0.002 |
| Multivariable-adjusted model 3 (+ BMI) | 1 (ref) | 0.89 (0.76 - 1.05) | 0.85 (0.71 - 1.01) | **0.70 (0.58 - 0.85)** |  | **0.89 (0.83 - 0.96)** | 9.15 | 0.002 |
| Multivariable-adjusted model 4  (+ red and processed meat; fully adjusted) | 1 (ref) | 0.89 (0.76 - 1.06) | 0.85 (0.72 - 1.01) | **0.71 (0.58 - 0.86)** |  | **0.89 (0.83 - 0.97)** | 8.39 | 0.004 |
| Estimates in bold indicate statistically significant results.  Minimally adjusted model had age as the underlying time variable and analyses were stratified by sex and age groups and adjusted for region of recruitment.  Multivariable-adjusted model 1: further adjusted for height, physical activity, Townsend deprivation index, education, employment status, ethnicity, diagnosis of diabetes, non-steroidal anti-inflammatory drug use, menopause status (women only), menopausal hormone therapy use (women only), and energy intake.  Multivariable-adjusted model 2: further adjusted for smoking status and alcohol intake in the multivariable adjusted model 1.  Multivariable-adjusted model 3: further adjusted for BMI from the multivariable adjusted model 2.  Multivariable-adjusted model 4 (fully adjusted model): further adjusted for red and processed meat intake reported at recruitment from the multivariable adjusted model 3.  Abbreviations: BMI, body mass index; Q, quantile; ref, reference group. | | | | | | | | |

| **Supplementary Table 5.** Hazard ratios and 95% confidence intervals for minimally adjusted and sequential adjustment models for total protein, protein from all dairy products, milk, and cheese, and total dietary calcium intake and risk of **breast cancer.** | | | | | | | | |
| --- | --- | --- | --- | --- | --- | --- | --- | --- |
| **Breast cancer** | Intakes of total protein, protein from all dairy products, milk, cheese, and dietary calcium | | | | | |  |  |
|  | Q1 | Q2 | Q3 | Q4 |  | Per 2.5% energy increase | χ^2^ | *P-trend* |
| **Total protein** |  |  |  |  |  |  |  |  |
| Minimally-adjusted model | 1 (ref) | 1.01 (0.89 - 1.14) | 0.95 (0.84 - 1.07) | 0.99 (0.88 - 1.12) |  | 0.99 (0.96 - 1.03) | 0.26 | 0.61 |
| Multivariable-adjusted model 1 | 1 (ref) | 1.02 (0.90 - 1.15) | 0.96 (0.84 - 1.09) | 0.99 (0.87 - 1.13) |  | 0.99 (0.95 - 1.03) | 0.31 | 0.58 |
| Multivariable-adjusted model 2  (+ smoking and alcohol intake) | 1 (ref) | 1.02 (0.90 - 1.15) | 0.96 (0.85 - 1.09) | 1.00 (0.87 - 1.13) |  | 0.99 (0.95 - 1.03) | 0.27 | 0.60 |
| Multivariable-adjusted model 3  (+ BMI; fully adjusted) | 1 (ref) | 1.02 (0.90 - 1.15) | 0.96 (0.84 - 1.08) | 0.99 (0.86 - 1.12) |  | 0.99 (0.95 - 1.02) | 0.47 | 0.49 |
| **Protein from dairy products** |  |  |  |  |  |  |  |  |
| Minimally-adjusted model | 1 (ref) | 1.03 (0.91 - 1.16) | 0.98 (0.86 - 1.11) | 0.92 (0.82 - 1.05) |  | 0.93 (0.86 - 1.01) | 3.07 | 0.08 |
| Multivariable-adjusted model 1 | 1 (ref) | 1.03 (0.92 - 1.17) | 0.98 (0.87 - 1.11) | 0.93 (0.82 - 1.06) |  | 0.94 (0.86 - 1.01) | 2.75 | 0.10 |
| Multivariable-adjusted model 2  (+ smoking and alcohol intake) | 1 (ref) | 1.04 (0.92 - 1.17) | 0.99 (0.87 - 1.12) | 0.93 (0.82 - 1.06) |  | 0.94 (0.87 - 1.01) | 2.67 | 0.10 |
| Multivariable-adjusted model 3  (+ BMI; fully adjusted) | 1 (ref) | 1.04 (0.92 - 1.17) | 0.99 (0.87 - 1.12) | 0.93 (0.82 - 1.06) |  | 0.94 (0.86 - 1.01) | 2.72 | 0.10 |
| **Protein from milk** |  |  |  |  |  |  |  |  |
| Minimally-adjusted model | 1 (ref) | 1.00 (0.89 - 1.13) | 0.98 (0.87 - 1.11) | 0.92 (0.82 - 1.05) |  | 0.90 (0.79 - 1.02) | 2.97 | 0.08 |
| Multivariable-adjusted model 1 | 1 (ref) | 1.00 (0.88 - 1.13) | 1.00 (0.88 - 1.13) | 0.94 (0.83 - 1.07) |  | 0.92 (0.81 - 1.04) | 1.90 | 0.17 |
| Multivariable-adjusted model 2  (+ smoking and alcohol intake) | 1 (ref) | 1.00 (0.89 - 1.13) | 1.00 (0.88 - 1.13) | 0.94 (0.83 - 1.07) |  | 0.91 (0.81 - 1.04) | 1.95 | 0.16 |
| Multivariable-adjusted model 3  (+ BMI; fully adjusted) | 1 (ref) | 1.00 (0.88 - 1.13) | 0.99 (0.88 - 1.12) | 0.94 (0.83 - 1.07) |  | 0.91 (0.80 - 1.03) | 2.09 | 0.15 |
| **Protein from cheese** |  |  |  |  |  |  |  |  |
| Minimally-adjusted model | 1 (ref) | 1.00 (0.88 - 1.13) | 1.04 (0.92 - 1.17) | 1.05 (0.93 - 1.18) |  | 1.04 (0.92 - 1.17) | 0.34 | 0.56 |
| Multivariable-adjusted model 1 | 1 (ref) | 1.00 (0.88 - 1.13) | 1.04 (0.92 - 1.18) | 1.04 (0.92 - 1.18) |  | 1.03 (0.91 - 1.16) | 0.19 | 0.66 |
| Multivariable-adjusted model 2  (+ smoking and alcohol intake) | 1 (ref) | 1.00 (0.88 - 1.14) | 1.04 (0.92 - 1.18) | 1.04 (0.92 - 1.18) |  | 1.02 (0.91 - 1.16) | 0.15 | 0.70 |
| Multivariable-adjusted model 3  (+ BMI; fully adjusted) | 1 (ref) | 1.00 (0.88 - 1.14) | 1.04 (0.92 - 1.18) | 1.04 (0.92 - 1.18) |  | 1.03 (0.91 - 1.16) | 0.21 | 0.65 |
|  |  |  |  |  |  |  |  |  |
| **Total dietary calcium** | Q1 | Q2 | Q3 | Q4 |  | Per 300 mg/day increase | χ^2^ | *P-trend* |
| Minimally-adjusted model | 1 (ref) | 0.95 (0.84 - 1.08) | 0.96 (0.85 - 1.08) | 0.95 (0.84 - 1.07) |  | 0.98 (0.93 - 1.02) | 1.00 | 0.32 |
| Multivariable-adjusted model 1 | 1 (ref) | 0.95 (0.84 - 1.08) | 0.95 (0.83 - 1.09) | 0.90 (0.78 - 1.05) |  | 0.95 (0.90 - 1.01) | 2.68 | 0.10 |
| Multivariable-adjusted model 2  (+ smoking and alcohol intake) | 1 (ref) | 0.96 (0.84 - 1.09) | 0.96 (0.84 - 1.10) | 0.91 (0.79 - 1.06) |  | 0.95 (0.90 - 1.01) | 2.35 | 0.12 |
| Multivariable-adjusted model 3  (+ BMI; fully adjusted) | 1 (ref) | 0.96 (0.84 - 1.09) | 0.96 (0.84 - 1.10) | 0.91 (0.79 - 1.06) |  | 0.96 (0.90 - 1.01) | 2.22 | 0.14 |
| Minimally adjusted model had age as the underlying time variable and analyses were stratified by age groups and adjusted for region of recruitment.  Multivariable-adjusted model 1: further adjusted for height, physical activity, Townsend deprivation index, education, employment, ethnicity, diagnosis of diabetes, menopausal hormone therapy use, oral contraceptive use, parity and age at first birth, age at menarche, and energy intake in the minimally adjusted model.  Multivariable-adjusted model 2: further adjusted for smoking status and alcohol intake in the multivariable adjusted model.  Multivariable-adjusted model 3 (fully adjusted model): further adjusted for BMI and menopause status from the multivariable adjusted model 2.  Abbreviations: BMI, body mass index; Q, quantile; ref, reference group. | | | | | | | | |

| **Supplementary Table 6.** Hazard ratios and 95% confidence intervals for minimally adjusted and sequential adjustment models for total protein, protein from all dairy products, milk, and cheese, and total dietary calcium intake and risk of **prostate cancer.** | | | | | | | | |
| --- | --- | --- | --- | --- | --- | --- | --- | --- |
| **Prostate cancer** | Intakes of total protein, protein from all dairy products, milk, cheese, and dietary calcium | | | | | |  |  |
|  | Q1 | Q2 | Q3 | Q4 |  | Per 2.5% energy increase | *χ*^2^ | *P-trend* |
| **Total protein** |  |  |  |  |  |  |  |  |
| Minimally-adjusted model | 1 (ref) | 1.07 (0.95 - 1.19) | 1.05 (0.93 - 1.17) | 0.96 (0.86 - 1.08) |  | 0.98 (0.95 - 1.02) | 0.71 | 0.40 |
| Multivariable-adjusted model 1 | 1 (ref) | 1.06 (0.95 - 1.19) | 1.05 (0.93 - 1.17) | 0.97 (0.86 - 1.10) |  | 0.99 (0.95 - 1.03) | 0.30 | 0.59 |
| Multivariable-adjusted model 2  (+ smoking and alcohol intake) | 1 (ref) | 1.06 (0.95 - 1.19) | 1.04 (0.93 - 1.17) | 0.98 (0.86 - 1.10) |  | 0.99 (0.95 - 1.03) | 0.28 | 0.59 |
| Multivariable-adjusted model 3  (+ BMI; fully adjusted) | 1 (ref) | 1.06 (0.95 - 1.19) | 1.05 (0.93 - 1.17) | 0.98 (0.87 - 1.11) |  | 0.99 (0.96 - 1.03) | 0.18 | 0.68 |
| **Protein from dairy products** |  |  |  |  |  |  |  |  |
| Minimally-adjusted model | 1 (ref) | 1.01 (0.89 - 1.13) | 1.08 (0.96 - 1.21) | 1.04 (0.92 - 1.16) |  | 1.03 (0.95 - 1.11) | 0.58 | 0.45 |
| Multivariable-adjusted model 1 | 1 (ref) | 1.00 (0.89 - 1.12) | 1.08 (0.96 - 1.21) | 1.04 (0.93 - 1.17) |  | 1.04 (0.96 - 1.12) | 0.80 | 0.37 |
| Multivariable-adjusted model 2  (+ smoking and alcohol intake) | 1 (ref) | 1.00 (0.89 - 1.12) | 1.08 (0.96 - 1.21) | 1.04 (0.93 - 1.17) |  | 1.04 (0.96 - 1.12) | 0.87 | 0.35 |
| Multivariable-adjusted model 3  (+ BMI; fully adjusted) | 1 (ref) | 1.00 (0.89 - 1.12) | 1.08 (0.96 - 1.21) | 1.04 (0.93 - 1.17) |  | 1.04 (0.96 - 1.12) | 0.85 | 0.36 |
| **Protein from milk** |  |  |  |  |  |  |  |  |
| Minimally-adjusted model | 1 (ref) | 1.04 (0.92 - 1.16) | 0.99 (0.89 - 1.12) | 1.10 (0.99 - 1.24) |  | 1.09 (0.97 - 1.23) | 1.96 | 0.16 |
| Multivariable-adjusted model 1 | 1 (ref) | 1.03 (0.92 - 1.16) | 1.00 (0.89 - 1.12) | 1.12 (1.00 - 1.26) |  | 1.11 (0.98 - 1.26) | 2.73 | 0.10 |
| Multivariable-adjusted model 2  (+ smoking and alcohol intake) | 1 (ref) | 1.03 (0.92 - 1.16) | 0.99 (0.88 - 1.12) | 1.12 (1.00 - 1.26) |  | 1.12 (0.98 - 1.27) | 2.88 | 0.09 |
| Multivariable-adjusted model 3  (+ BMI; fully adjusted) | 1 (ref) | 1.03 (0.92 - 1.15) | 0.99 (0.88 - 1.11) | 1.12 (1.00 - 1.26) |  | 1.11 (0.98 - 1.26) | 2.74 | 0.10 |
| **Protein from cheese** |  |  |  |  |  |  |  |  |
| Minimally-adjusted model | 1 (ref) | 1.01 (0.90 - 1.13) | 1.04 (0.93 - 1.16) | 0.97 (0.87 - 1.09) |  | 0.95 (0.85 - 1.07) | 0.63 | 0.43 |
| Multivariable-adjusted model 1 | 1 (ref) | 0.99 (0.88 - 1.11) | 1.03 (0.92 - 1.15) | 0.96 (0.86 - 1.08) |  | 0.95 (0.85 - 1.08) | 0.60 | 0.44 |
| Multivariable-adjusted model 2  (+ smoking and alcohol intake) | 1 (ref) | 0.99 (0.88 - 1.11) | 1.03 (0.92 - 1.15) | 0.96 (0.86 - 1.08) |  | 0.96 (0.85 - 1.08) | 0.56 | 0.46 |
| Multivariable-adjusted model 3  (+ BMI; fully adjusted) | 1 (ref) | 0.99 (0.88 - 1.11) | 1.03 (0.92 - 1.15) | 0.97 (0.86 - 1.08) |  | 0.96 (0.85 - 1.08) | 0.52 | 0.47 |
|  |  |  |  |  |  |  |  |  |
| **Total dietary calcium** | Q1 | Q2 | Q3 | Q4 |  | Per 300 mg/day increase | *χ*^2^ | *P-trend* |
| Minimally-adjusted model | 1 (ref) | **1.12 (1.00 - 1.25)** | **1.12 (1.00 - 1.26)** | **1.14 (1.02 - 1.28)** |  | 1.04 (0.99 - 1.08) | 2.85 | 0.09 |
| Multivariable-adjusted model 1 | 1 (ref) | **1.12 (1.00 - 1.27)** | 1.12 (0.99 - 1.27) | **1.15 (1.01 - 1.32)** |  | 1.04 (0.99 - 1.09) | 1.99 | 0.16 |
| Multivariable-adjusted model 2  (+ smoking and alcohol intake) | 1 (ref) | **1.13 (1.00 - 1.27)** | 1.13 (0.99 - 1.28) | **1.16 (1.01 - 1.33)** |  | 1.04 (0.99 - 1.09) | 2.14 | 0.14 |
| Multivariable-adjusted model 3  (+ BMI; fully adjusted) | 1 (ref) | **1.12 (1.00 - 1.27)** | 1.13 (0.99 - 1.28) | **1.16 (1.01 - 1.33)** |  | 1.04 (0.99 - 1.09) | 2.05 | 0.15 |
| Estimates in bold indicate statistically significant results.  Minimally adjusted model had age as the underlying time variable and analyses were stratified by sex and age groups and adjusted for region of recruitment.  Multivariable-adjusted model 1: further adjusted for height, physical activity, Townsend deprivation index, education, ethnicity, diabetes status, marital status, and energy intake from the minimally adjusted model.  Multivariable-adjusted model 2: further adjusted for smoking status and alcohol intake in the multivariable adjusted model.  Multivariable-adjusted model 3 (fully adjusted model): further adjusted for BMI in the multivariable adjusted model 2.  Abbreviations: BMI, body mass index; Q, quantile; ref, reference group. | | | | | | | | |

| **Supplementary Table 7.** Multivariable-adjusted hazard ratios (95% CI) for colon and rectal cancer by intake of total protein, protein from all dairy products, milk, and cheese, and total dietary calcium. | | | | |
| --- | --- | --- | --- | --- |
| **Total protein, dairy protein, and calcium intake** | **Colon cancer (n=730)** | **Rectal cancer (n=395)** | 𝛘^2^ | **p-value** |
| Total protein | 0.98 (0.92 - 1.05) | 0.99 (0.90 - 1.09) | 0.04 | 0.84 |
| Protein from dairy | 0.81 (0.70 - 0.93) | 0.82 (0.67 - 0.99) | 0.01 | 0.94 |
| Protein from milk | 0.80 (0.63 - 1.00) | 0.68 (0.50 - 0.94) | 0.58 | 0.45 |
| Protein from cheese | 0.80 (0.64 - 1.00) | 1.03 (0.78 - 1.38) | 1.92 | 0.17 |
| Total dietary calcium | 0.91 (0.82 - 1.01) | 0.90 (0.79 - 1.03) | 0.01 | 0.94 |
| Total protein and dairy protein are modeled as a 2.5% energy increase whereas total dietary calcium intake is modelled as a 300 mg/day increase.  All models used age as the underlying time variable, were stratified by sex and age groups at recruitment, and further adjusted for height, physical activity, Townsend deprivation index, education, employment status, smoking status, alcohol intake, ethnicity, diagnosis of diabetes, BMI, menopause status (women only), menopausal hormone therapy use (women only), red and processed meat intake, non-steroidal anti-inflammatory drug use, and energy intake.  χ^2^ and p-value for heterogeneity represent Wald’s test for heterogeneity between tumour subgroups and protein intake (per 2.5% intake) or calcium intake per 300 mg/day. | | | | |

| **Supplementary Table 8.** Hazard ratios (95% CI) for intake of total protein, protein from dairy products and dairy sources, and total dietary calcium with colorectal, breast, and prostate cancer with **additional adjustments for other dietary factors.** | | | | | | | | |
| --- | --- | --- | --- | --- | --- | --- | --- | --- |
|  | Intakes of total protein, protein from dairy products, and calcium | | | |  |  |  |  |
| **Colorectal cancer** | Q1 | Q2 | Q3 | Q4 |  | Incremental increase^1^ | χ^2^ | *P-trend* |
| **Total protein** |  |  |  |  |  |  |  |  |
| Fully adjusted model | 1 (ref) | 1.13 (0.96 - 1.32) | 1.00 (0.85 - 1.18) | 0.99 (0.83 - 1.18) |  | 0.98 (0.93 - 1.03) | 0.79 | 0.37 |
| Fully adjusted model + dietary factors | 1 (ref) | 1.14 (0.97 - 1.33) | 1.02 (0.86 - 1.20) | 1.01 (0.85 - 1.21) |  | 0.98 (0.93 - 1.04) | 0.35 | 0.56 |
| **Protein from dairy products** |  |  |  |  |  |  |  |  |
| Fully adjusted model | 1 (ref) | 0.91 (0.78 - 1.06) | 0.88 (0.75 - 1.03) | **0.80 (0.67 - 0.94)** |  | **0.82 (0.74 - 0.92)** | 11.61 | 0.001 |
| Fully adjusted model + dietary factors | 1 (ref) | 0.91 (0.78 - 1.06) | 0.88 (0.75 - 1.03) | **0.80 (0.67 - 0.94)** |  | **0.83 (0.74 - 0.93)** | 10.43 | 0.001 |
| **Protein from milk** |  |  |  |  |  |  |  |  |
| Fully adjusted model | 1 (ref) | 0.87 (0.74 - 1.01) | **0.80 (0.68 - 0.93)** | **0.79 (0.67 - 0.94)** |  | **0.76 (0.63 - 0.91)** | 9.01 | 0.003 |
| Fully adjusted model + dietary factors | 1 (ref) | 0.86 (0.74 - 1.01) | **0.79 (0.67 - 0.93)** | **0.79 (0.67 - 0.93)** |  | **0.77 (0.64 - 0.92)** | 8.02 | 0.005 |
| **Protein from cheese** |  |  |  |  |  |  |  |  |
| Fully adjusted model | 1 (ref) | 0.91 (0.77 - 1.07) | 0.95 (0.82 - 1.12) | 0.89 (0.76 - 1.05) |  | 0.91 (0.76 - 1.08) | 1.25 | 0.26 |
| Fully adjusted model + dietary factors | 1 (ref) | 0.91 (0.77 - 1.07) | 0.95 (0.82 - 1.12) | 0.89 (0.76 - 1.05) |  | 0.90 (0.76 - 1.07) | 1.33 | 0.25 |
| **Total dietary calcium** |  |  |  |  |  |  |  |  |
| Fully adjusted model | 1 (ref) | 0.89 (0.76 - 1.06) | 0.85 (0.72 - 1.01) | **0.71 (0.58 - 0.86)** |  | **0.89 (0.83 - 0.97)** | 8.39 | 0.004 |
| Fully adjusted model + dietary factors | 1 (ref) | 0.91 (0.77 - 1.07) | 0.87 (0.73 - 1.04) | **0.73 (0.60 - 0.89)** |  | **0.91 (0.84 - 0.98)** | 6.34 | 0.012 |
|  |  |  |  |  |  |  |  |  |
| **Breast cancer** | Q1 | Q2 | Q3 | Q4 |  | Incremental increase^1^ | χ^2^ | *P-trend* |
| **Total protein** |  |  |  |  |  |  |  |  |
| Fully adjusted model | 1 (ref) | 1.02 (0.90 - 1.15) | 0.96 (0.84 - 1.08) | 0.99 (0.86 - 1.12) |  | 0.99 (0.95 - 1.02) | 0.47 | 0.49 |
| Fully adjusted model + dietary factors | 1 (ref) | 1.01 (0.89 - 1.14) | 0.95 (0.84 - 1.09) | 0.98 (0.86 - 1.13) |  | 0.99 (0.95 - 1.03) | 0.46 | 0.50 |
| **Protein from dairy products** |  |  |  |  |  |  |  |  |
| Fully adjusted model | 1 (ref) | 1.04 (0.92 - 1.17) | 0.99 (0.87 - 1.12) | 0.93 (0.82 - 1.06) |  | 0.94 (0.86 - 1.01) | 2.72 | 0.10 |
| Fully adjusted model + dietary factors | 1 (ref) | 1.03 (0.91 - 1.17) | 0.98 (0.87 - 1.12) | 0.93 (0.82 - 1.06) |  | 0.93 (0.86 - 1.01) | 2.94 | 0.09 |
| **Protein from milk** |  |  |  |  |  |  |  |  |
| Fully adjusted model | 1 (ref) | 1.00 (0.88 - 1.13) | 0.99 (0.88 - 1.12) | 0.94 (0.83 - 1.07) |  | 0.91 (0.80 - 1.03) | 2.09 | 0.15 |
| Fully adjusted model + dietary factors | 1 (ref) | 0.99 (0.87 - 1.12) | 0.98 (0.87 - 1.12) | 0.92 (0.81 - 1.04) |  | 0.89 (0.79 - 1.01) | 3.15 | 0.08 |
|  |  |  |  |  |  |  |  |  |
| **Protein from cheese** |  |  |  |  |  |  |  |  |
| Fully adjusted model | 1 (ref) | 1.00 (0.88 - 1.14) | 1.04 (0.92 - 1.18) | 1.04 (0.92 - 1.18) |  | 1.03 (0.91 - 1.16) | 0.21 | 0.65 |
| Fully adjusted model + dietary factors | 1 (ref) | 0.99 (0.87 - 1.13) | 1.03 (0.91 - 1.17) | 1.04 (0.92 - 1.18) |  | 1.03 (0.91 - 1.17) | 0.24 | 0.62 |
| **Total dietary calcium** |  |  |  |  |  |  |  |  |
| Fully adjusted model | 1 (ref) | 0.96 (0.84 - 1.09) | 0.96 (0.84 - 1.10) | 0.91 (0.79 - 1.06) |  | 0.96 (0.90 - 1.01) | 2.22 | 0.14 |
| Fully adjusted model + dietary factors | 1 (ref) | 0.95 (0.84 - 1.08) | 0.96 (0.84 - 1.10) | 0.92 (0.79 - 1.07) |  | 0.96 (0.90 - 1.02) | 1.89 | 0.17 |
|  |  |  |  |  |  |  |  |  |
| **Prostate cancer** | Q1 | Q2 | Q3 | Q4 |  | Incremental increase^1^ | χ^2^ | *P-trend* |
| **Total protein** |  |  |  |  |  |  |  |  |
| Fully adjusted model | 1 (ref) | 1.06 (0.95 - 1.19) | 1.05 (0.93 - 1.17) | 0.98 (0.87 - 1.11) |  | 0.99 (0.96 - 1.03) | 0.18 | 0.68 |
| Fully adjusted model + dietary factors | 1 (ref) | 1.04 (0.93 - 1.17) | 1.02 (0.91 - 1.14) | 0.95 (0.84 - 1.08) |  | 0.98 (0.95 - 1.02) | 0.69 | 0.40 |
| **Protein from dairy products** |  |  |  |  |  |  |  |  |
| Fully adjusted model | 1 (ref) | 1.00 (0.89 - 1.12) | 1.08 (0.96 - 1.21) | 1.04 (0.93 - 1.17) |  | 1.04 (0.96 - 1.12) | 0.85 | 0.36 |
| Fully adjusted model + dietary factors | 1 (ref) | 1.00 (0.88 - 1.12) | 1.07 (0.95 - 1.20) | 1.04 (0.92 - 1.17) |  | 1.04 (0.96 - 1.12) | 0.85 | 0.36 |
| **Protein from milk** |  |  |  |  |  |  |  |  |
| Fully adjusted model | 1 (ref) | 1.03 (0.92 - 1.15) | 0.99 (0.88 - 1.11) | **1.12 (1.00 - 1.26)** |  | 1.11 (0.98 - 1.26) | 2.74 | 0.10 |
| Fully adjusted model + dietary factors | 1 (ref) | 1.02 (0.91 - 1.14) | 0.98 (0.87 - 1.10) | 1.10 (0.98 - 1.24) |  | 1.09 (0.96 - 1.24) | 1.92 | 0.17 |
| **Protein from cheese** |  |  |  |  |  |  |  |  |
| Fully adjusted model | 1 (ref) | 0.99 (0.88 - 1.11) | 1.03 (0.92 - 1.15) | 0.97 (0.86 - 1.08) |  | 0.96 (0.85 - 1.08) | 0.52 | 0.47 |
| Fully adjusted model + dietary factors | 1 (ref) | 0.98 (0.88 - 1.10) | 1.03 (0.92 - 1.15) | 0.97 (0.87 - 1.09) |  | 0.97 (0.86 - 1.09) | 0.30 | 0.59 |
| **Total dietary calcium** |  |  |  |  |  |  |  |  |
| Fully adjusted model | 1 (ref) | **1.12 (1.00 - 1.27)** | 1.13 (0.99 - 1.28) | **1.16 (1.01 - 1.33)** |  | 1.04 (0.99 - 1.09) | 2.05 | 0.15 |
| Fully adjusted model + dietary factors | 1 (ref) | 1.12 (0.99 - 1.26) | 1.12 (0.98 - 1.27) | **1.16 (1.01 - 1.34)** |  | 1.04 (0.99 - 1.09) | 2.07 | 0.15 |
| ^1^ For total protein, protein from dairy products, protein from milk, and protein from cheese, an incremental increase represents a 2.5% higher energy intake per day whereas for total dietary calcium this represents a 300 mg/day increase.  Estimates in bold indicate statistically significant results.  All models used age as the underlying time variable, were stratified by age groups at recruitment, and further adjusted for height, physical activity, Townsend deprivation index, education, employment status, smoking status, alcohol intake, ethnicity, diagnosis of diabetes, BMI, energy intake.  For colorectal cancer analyses: all models were stratified by sex and further adjusted for menopause status (women only), menopausal hormone therapy use (women only), red and processed meat intake, and non-steroidal anti-inflammatory drug use.  For breast cancer analyses: models were further adjusted for menopausal hormone therapy use, oral contraceptive use, parity and age at first birth, age at menarche, BMI and menopause status.  For prostate cancer analyses: models were further adjusted for marital status.  Fully adjusted model + dietary factors were further adjusted for fruit and vegetable intake and fibre intake as well as red and processed meat intake for breast cancer and prostate cancer analyses.  Abbreviations: BMI, body mass index; Q, quantile; ref, reference group. | | | | | | | | |

| **Supplementary Table 9.** Hazard ratios (95% CI) for intake of total protein, protein from dairy and dairy sources, and total dietary calcium with risk of colorectal, breast, and prostate cancer for participants who completed a minimum of **three 24-hour dietary assessments.** | | | | | |
| --- | --- | --- | --- | --- | --- |
|  | Intakes of total protein, protein from dairy products, and total dietary calcium | | | | |
| **Colorectal cancer**  (734 cases, 69,223 participants) | Q1 | Q2 | Q3 | Q4 | Incremental increase^1^ |
| **Total protein** | 1 (ref) | 0.92 (0.73 - 1.16) | 0.99 (0.78 - 1.26) | 0.97 (0.74 - 1.27) | 1.00 (0.93 - 1.07) |
| **Protein from dairy** | 1 (ref) | 0.88 (0.72 - 1.08) | 0.89 (0.73 - 1.09) | **0.75 (0.61 - 0.94)** | **0.79 (0.68 - 0.92)** |
| **Protein from milk** | 1 (ref) | 0.85 (0.69 - 1.03) | **0.74 (0.61 - 0.91)** | **0.79 (0.64 - 0.98)** | **0.75 (0.59 - 0.95)** |
| **Protein from cheese** | 1 (ref) | 0.96 (0.79 - 1.18) | 0.91 (0.74 - 1.11) | 0.92 (0.74 - 1.13) | 0.87 (0.69 - 1.10) |
| **Total dietary calcium** | 1 (ref) | **0.77 (0.62 - 0.95)** | **0.78 (0.62 - 0.97)** | **0.71 (0.56 - 0.91)** | **0.89 (0.80 - 0.99)** |
|  |  |  |  |  |  |
| **Breast cancer**  (1,187 cases, 38,282 participants) | Q1 | Q2 | Q3 | Q4 | Incremental increase^1^ |
| **Total protein** | 1 (ref) | 1.03 (0.88 - 1.21) | 0.92 (0.78 - 1.08) | 0.96 (0.81 - 1.14) | 0.98 (0.93 - 1.04) |
| **Protein from dairy** | 1 (ref) | 1.06 (0.90 - 1.25) | 1.01 (0.86 - 1.19) | 0.99 (0.84 - 1.17) | 0.97 (0.87 - 1.08) |
| **Protein from milk** | 1 (ref) | 0.99 (0.85 - 1.17) | 0.93 (0.79 - 1.10) | 0.96 (0.81 - 1.13) | 0.91 (0.77 - 1.08) |
| **Protein from cheese** | 1 (ref) | 0.94 (0.79 - 1.11) | 1.08 (0.92 - 1.26) | 1.03 (0.88 - 1.21) | 1.03 (0.87 - 1.22) |
| **Total dietary calcium** | 1 (ref) | 1.01 (0.85 - 1.19) | 0.98 (0.82 - 1.17) | 0.96 (0.79 - 1.16) | 0.95 (0.88 - 1.04) |
|  |  |  |  |  |  |
| **Prostate cancer**  (1,451 cases, 30,941 participants) | Q1 | Q2 | Q3 | Q4 | Incremental increase^1^ |
| **Total protein** | 1 (ref) | 1.09 (0.94 - 1.26) | 1.10 (0.95 - 1.28) | 0.99 (0.84 - 1.16) | 1.00 (0.95 - 1.05) |
| **Protein from dairy** | 1 (ref) | 0.99 (0.85 - 1.15) | 1.05 (0.90 - 1.22) | 1.06 (0.91 - 1.23) | 1.07 (0.96 - 1.19) |
| **Protein from milk** | 1 (ref) | 1.04 (0.89 - 1.21) | 1.05 (0.90 - 1.22) | 1.11 (0.95 - 1.29) | 1.12 (0.95 - 1.32) |
| **Protein from cheese** | 1 (ref) | 0.90 (0.77 - 1.04) | 0.99 (0.86 - 1.15) | 0.96 (0.83 - 1.12) | 0.99 (0.84 - 1.17) |
| **Total dietary calcium** | 1 (ref) | **1.18 (1.01 - 1.37)** | 1.15 (0.97 - 1.35) | **1.19 (1.00 - 1.43)** | 1.03 (0.97 - 1.11) |
| ^1^ For total protein, protein from dairy products, protein from milk, and protein from cheese, an incremental increase represents a 2.5% higher energy intake per day whereas for total dietary calcium this represents a 300 mg/day increase.  Estimates in bold indicate statistically significant results.  All models used age as the underlying time variable and were stratified by age groups at recruitment, and further adjusted for height, physical activity, Townsend deprivation index, education, employment status, smoking status, alcohol intake, ethnicity, diagnosis of diabetes, BMI, and energy intake.  For colorectal cancer analyses: all models were stratified by sex and further adjusted for menopause status (women only), menopausal hormone therapy use (women only), red and processed meat intake, and non-steroidal anti-inflammatory drug use.  For breast cancer analyses: models were further adjusted for menopausal hormone therapy use, oral contraceptive use, parity and age at first birth, age at menarche, BMI and menopause status.  For prostate cancer analyses: models were further adjusted for marital status.  Abbreviations: BMI, body mass index; Q, quantile. | | | | | |

| **Supplementary Table 10.** Hazard ratios (95% CI) for intake of total protein, protein from dairy products and dairy sources, and total dietary calcium with risk of colorectal, breast, and prostate cancer **removing the first two years of follow-up.** | | | | | |
| --- | --- | --- | --- | --- | --- |
|  | Intakes of total protein, protein from dairy products, and total dietary calcium | | | |  |
| **Colorectal cancer**  (978 cases, 111,724 participants) | Q1 | Q2 | Q3 | Q4 | Incremental increase^1^ |
| **Total protein** | 1 (ref) | 1.10 (0.93 - 1.31) | 0.96 (0.80 - 1.16) | 0.99 (0.81 - 1.20) | 0.97 (0.91 - 1.03) |
| **Protein from dairy** | 1 (ref) | 0.89 (0.75 - 1.05) | 0.87 (0.73 - 1.04) | **0.80 (0.66 - 0.96)** | **0.83 (0.74 - 0.94)** |
| **Protein from milk** | 1 (ref) | 0.89 (0.75 - 1.05) | **0.78 (0.66 - 0.94)** | **0.80 (0.66 - 0.96)** | **0.76 (0.62 - 0.92)** |
| **Protein from cheese** | 1 (ref) | 0.89 (0.75 - 1.07) | 0.93 (0.78 - 1.11) | 0.90 (0.75 - 1.07) | 0.92 (0.76 - 1.11) |
| **Total dietary calcium** | 1 (ref) | 0.90 (0.75 - 1.09) | 0.84 (0.70 - 1.02) | **0.70 (0.57 - 0.87)** | **0.89 (0.82 - 0.97)** |
|  |  |  |  |  |  |
| **Breast cancer**  (1,598 cases, 61,756 participants) | Q1 | Q2 | Q3 | Q4 | Incremental increase^1^ |
| **Total protein** | 1 (ref) | 1.00 (0.87 - 1.14) | 0.87 (0.76 - 1.01) | 0.95 (0.83 - 1.10) | 0.97 (0.93 - 1.01) |
| **Protein from dairy** | 1 (ref) | 1.04 (0.91 - 1.20) | 0.99 (0.86 - 1.14) | 0.95 (0.82 - 1.09) | 0.95 (0.87 - 1.04) |
| **Protein from milk** | 1 (ref) | 0.95 (0.83 - 1.10) | 1.02 (0.89 - 1.18) | 0.93 (0.81 - 1.08) | 0.92 (0.80 - 1.06) |
| **Protein from cheese** | 1 (ref) | 0.99 (0.86 - 1.15) | 1.05 (0.92 - 1.21) | 1.07 (0.93 - 1.23) | 1.05 (0.91 - 1.21) |
| **Total dietary calcium** | 1 (ref) | 0.93 (0.81 - 1.07) | 0.94 (0.81 - 1.10) | 0.93 (0.79 - 1.10) | 0.96 (0.90 - 1.03) |
|  |  |  |  |  |  |
| **Prostate cancer**  (1,976 cases, 49,968 participants) | Q1 | Q2 | Q3 | Q4 | Incremental increase^1^ |
| **Total protein** | 1 (ref) | 1.11 (0.98 - 1.26) | 1.04 (0.92 - 1.18) | 1.02 (0.89 - 1.17) | 0.99 (0.95 - 1.03) |
| **Protein from dairy** | 1 (ref) | 0.97 (0.85 - 1.10) | 1.07 (0.94 - 1.21) | 1.02 (0.90 - 1.16) | 1.04 (0.95 - 1.13) |
| **Protein from milk** | 1 (ref) | 1.02 (0.90 - 1.16) | 0.99 (0.87 - 1.13) | 1.12 (0.98 - 1.27) | 1.09 (0.95 - 1.26) |
| **Protein from cheese** | 1 (ref) | 0.99 (0.87 - 1.13) | 1.02 (0.91 - 1.16) | 0.96 (0.85 - 1.09) | 0.95 (0.83 - 1.09) |
| **Total dietary calcium** | 1 (ref) | 1.12 (0.98 - 1.28) | 1.11 (0.97 - 1.28) | 1.13 (0.97 - 1.32) | 1.02 (0.97 - 1.08) |
| ^1^ For total protein, protein from dairy products, protein from milk, and protein from cheese, an incremental increase represents a 2.5% higher energy intake per day whereas for total dietary calcium this represents a 300 mg/day increase.  Estimates in bold indicate statistically significant results.  All models used age as the underlying time variable, were stratified by age groups at recruitment, and further adjusted for height, physical activity, Townsend deprivation index, education, employment status, smoking status, alcohol intake, ethnicity, diagnosis of diabetes, BMI, energy intake.  For colorectal cancer analyses: all models were stratified by sex and further adjusted for menopause status (women only), menopausal hormone therapy use (women only), red and processed meat intake, and non-steroidal anti-inflammatory drug use.  For breast cancer analyses: models were further adjusted for menopausal hormone therapy use, oral contraceptive use, parity and age at first birth, age at menarche, BMI and menopause status.  For prostate cancer analyses: models were further adjusted for marital status.  Abbreviations: BMI, body mass index; Q, quantile. | | | | | |

# Supplementary Figures

Number of 24-hour dietary assessments completed by participants:

- Two: 44,994
- Three: 38,480
- Four: 26,032
- Five: 4,711

Recruitment

Oxford WebQ 24-hour dietary assessment

First

(n=31,832)

Second (n=75,549)

Fourth (n=82,514)

Fifth

(n=76,645)

Third (n=66,571)

WebQ subsample: completed a minimum of two WebQs (n=114,217)

2006 -2009

End of recruitment

2012

2010

2011

## **Supplementary Figure 1.** Dietary assessment for participants in the UK Biobank over time.

Numbers for 24-hour dietary assessments exclude participants who withdrew their consent, who were diagnosed with a prevalent cancer at recruitment, who did not complete a minimum of two valid 24-hour dietary assessments (24-hour dietary assessments were deemed invalid if they reported extreme energy intake or they reported that they were ill or fasting on the respective day), or who were censored before they completed their final 24-hour dietary assessment.

**30,747 participants excluded:**

• **922** participants withdrew consent/ineligible

• **321** Genetic sex did not match reported sex

• **29,504** participants diagnosed with cancer (excluding non-melanoma skin cancer)

**503,317 participants recruited to UK Biobank**

**472,570 participants**

**251,938 participants excluded:**

• 251,938 participants did not complete a 24-hour dietary assessment

**3,031 participants excluded:**

• **2439** were excluded after removing 24-hour dietary assessments with extreme energy values

• **592** were excluded after removing 24-hour dietary assessments where participants reported they were ill or fasting

**220,632 participants**

**217,601 participants**

**100,487 participants excluded:**

• 100,487 participants did not complete a minimum of two valid 24-hour dietary assessments

**2,897 participants excluded:**

• 2,897 participants were left censored and excluded before the completion of their final 24-hour dietary assessment

**117,114 participants**

**114,217 participants**

**51,278 men**

**62,939 women**

## **Supplementary Figure 2.** Flow diagram showing eligible participants for this study.

## **
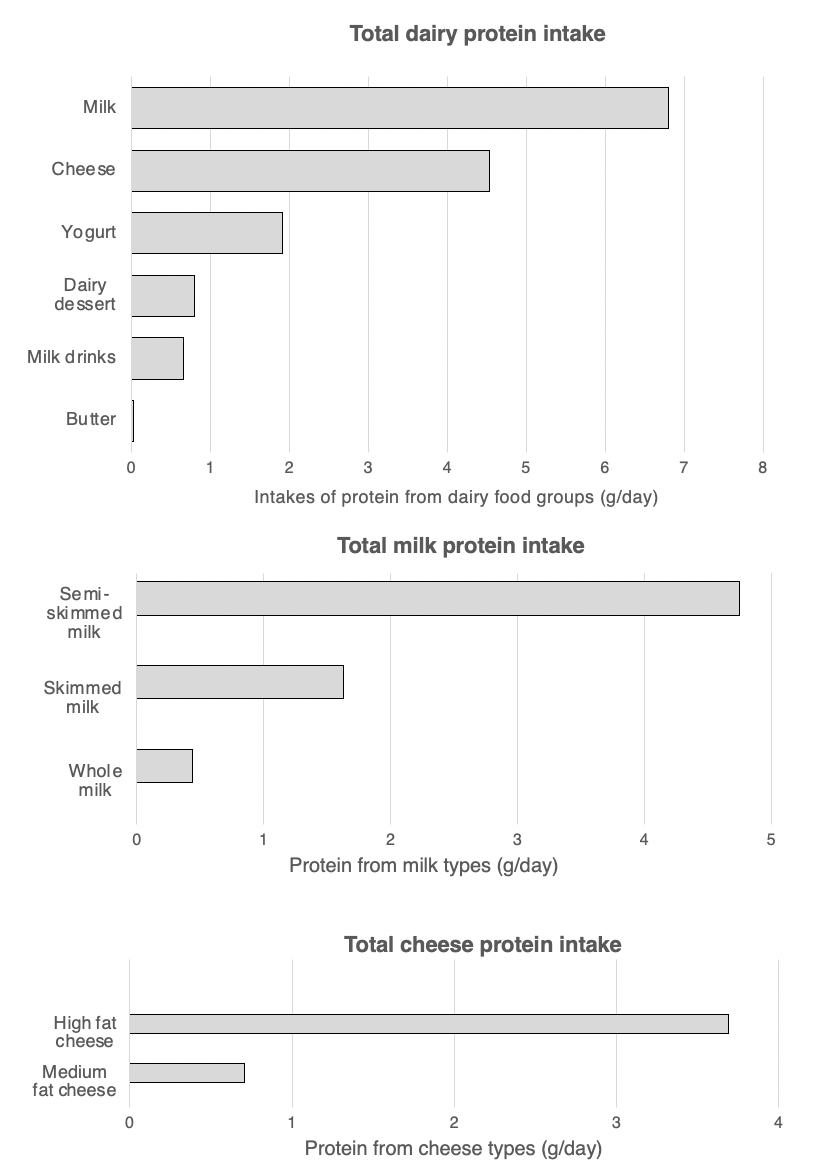
Supplementary Figure 3.** Dairy food group (g/day of protein) contributors for total dairy protein, milk protein, and cheese protein intake.

##

## **Supplementary Figure 4.** Multivariable-adjusted hazard ratios (95% CI) for colorectal, breast and prostate cancer risk by **total** **grams** of dairy products, milk, and cheese.

All models used age as the underlying time variable, were stratified by age groups at recruitment, and further adjusted for height, physical activity, Townsend deprivation index, education, employment status, smoking status, alcohol intake, ethnicity, diagnosis of diabetes, BMI, energy intake.

For colorectal cancer analyses: all models were stratified by sex and further adjusted for menopause status (women only), menopausal hormone therapy use (women only), red and processed meat intake, and non-steroidal anti-inflammatory drug use.

For breast cancer analyses: models were further adjusted for menopausal hormone therapy use, oral contraceptive use, parity and age at first birth, age at menarche, BMI and menopause status.

For prostate cancer analyses: models were further adjusted for marital status.

Abbreviations: BMI, body mass index; HR, hazard ratio; g/day, grams per day; CI, confidence interval; N, number of participants; Q, quantile.

## **Supplementary Figure 5.** Intake of total protein from per 2.5% energy increase by subgroups and risk of colorectal cancer, breast cancer, prostate cancer.

All models used age as the underlying time variable, were stratified by age groups at recruitment, and further adjusted for height, physical activity, Townsend deprivation index, education, employment status, smoking status (except when smoking status was subgroup if interest), alcohol intake (except when alcohol was the subgroup of interest), ethnicity, diagnosis of diabetes, BMI (except when BMI was subgroup of interest), and energy intake.

For colorectal cancer analyses: all models were stratified by sex (except when sex was subgroup of interest) and further adjusted for menopause status (women only) and menopausal hormone therapy use (women only), red and processed meat intake, non-steroidal anti-inflammatory drug use.

For breast cancer analyses: models were adjusted for menopausal hormone therapy use, oral contraceptive use, parity and age at first birth, age at menarche, BMI and menopause status (except when menopause status was subgroup of interest).

For prostate cancer analyses: models were further adjusted for marital status.

χ^2^ represents improvement of model fit and p-value represents heterogeneity between subgroups obtained from likelihood ratio tests for including an interaction term between subgroup of interest and total protein (modelled as a 2.5% energy increase) into the model.

Abbreviations: BMI, body mass index; CI, confidence interval; g/day, grams per day; HR, hazard ratio; kg/m^2^, kilograms per metre squared; N, number of participants.

## **Supplementary Figure 6.** Intake of protein from total dairy products per 2.5% energy increase by subgroups and risk of colorectal cancer, breast cancer, prostate cancer.

All models used age as the underlying time variable, were stratified by age groups at recruitment, and further adjusted for height, physical activity, Townsend deprivation index, education, employment status, smoking status (except when smoking status was subgroup if interest), alcohol intake (except when alcohol was the subgroup of interest), ethnicity, diagnosis of diabetes, BMI (except when BMI was subgroup of interest), and energy intake.

For colorectal cancer analyses: all models were stratified by sex (except when sex was subgroup of interest) and further adjusted for menopause status (women only) and menopausal hormone therapy use (women only), red and processed meat intake, non-steroidal anti-inflammatory drug use.

For breast cancer analyses: models were adjusted for menopausal hormone therapy use, oral contraceptive use, parity and age at first birth, age at menarche, BMI and menopause status (except when menopause status was subgroup of interest).

For prostate cancer analyses: models were further adjusted for marital status.

χ^2^ represents improvement of model fit and p-value represents heterogeneity between subgroups obtained from likelihood ratio tests for including an interaction term between subgroup of interest and all dairy protein (modelled as a 2.5% energy increase) into the model.

Abbreviations: BMI, body mass index; CI, confidence interval; g/day, grams per day; HR, hazard ratio; kg/m^2^, kilograms per metre squared; N, number of participants.

## **Supplementary Figure 7.** Intake of milk protein per 2.5% energy increase by subgroups risk of colorectal cancer, breast cancer, prostate cancer.

All models used age as the underlying time variable and were stratified by age groups at recruitment, and further adjusted for height, physical activity, Townsend deprivation index, education, employment status, smoking status (except when smoking status was subgroup if interest), alcohol intake (except when alcohol was the subgroup of interest), ethnicity, diagnosis of diabetes, body mass index (except when body mass index was subgroup of interest), and energy intake.

For colorectal cancer analyses: all models were stratified by sex (except when sex was subgroup of interest) and further adjusted for menopause status (women only), menopausal hormone therapy use (women only), red and processed meat intake, and non-steroidal anti-inflammatory drug use.

For breast cancer analyses: models were further adjusted for menopausal hormone therapy use, oral contraceptive use, parity and age at first birth, age at menarche, BMI and menopause status (except when menopause status was subgroup of interest).

For prostate cancer analyses: models were further adjusted for marital status.

χ^2^ represents improvement of model fit and p-value represents heterogeneity between subgroups obtained from likelihood ratio tests for including an interaction term between subgroup of interest and milk protein (modelled as a 2.5% energy increase) into the model.

Abbreviations: BMI, body mass index; CI, confidence interval; g/day, grams per day; HR, hazard ratio; kg/m^2^, kilograms per metre squared; N, number of participants.

**Supplementary Figure 8**. Intake of cheese protein per 2.5% energy increase by subgroups risk of colorectal cancer, breast cancer, prostate cancer.

All models used age as the underlying time variable, were stratified by age groups at recruitment, and further adjusted for height, physical activity, Townsend deprivation index, education, employment status, smoking status (except when smoking status was subgroup if interest), alcohol intake (except when alcohol was the subgroup of interest), ethnicity, diagnosis of diabetes, BMI (except when BMI was subgroup of interest), and energy intake.

For colorectal cancer analyses: all models were stratified by sex (except when sex was subgroup of interest) and further adjusted for menopause status (women only) and menopausal hormone therapy use (women only), red and processed meat intake, non-steroidal anti-inflammatory drug use.

For breast cancer analyses: models were further adjusted for menopausal hormone therapy use, oral contraceptive use, parity and age at first birth, age at menarche, BMI and menopause status (except when menopause status was subgroup of interest).

For prostate cancer analyses: models were further adjusted for marital status.

χ^2^ represents improvement of model fit and p-value represents heterogeneity between subgroups obtained from likelihood ratio tests for including an interaction term between subgroup of interest and cheese protein (modelled as a 2.5% energy increase) into the model.

Abbreviations: BMI, body mass index; CI, confidence interval; g/day, grams per day; HR, hazard ratio; kg/m^2^, kilograms per metre squared; N, number of participants.

**Supplementary Figure 9.** Intake of dietary calcium per 300 mg/day increase by subgroups risk of colorectal cancer, breast cancer, prostate cancer.

All models used age as the underlying time variable, were stratified by age groups at recruitment, and further adjusted for height, physical activity, Townsend deprivation index, education, employment status, smoking status (except when smoking status was subgroup if interest), alcohol intake (except when alcohol was the subgroup of interest), ethnicity, diagnosis of diabetes, BMI (except when BMI was subgroup of interest), and energy intake.

For colorectal cancer analyses: all models were stratified by sex (except when sex was subgroup of interest) and further adjusted for menopause status (women only) and menopausal hormone therapy use (women only), red and processed meat intake, non-steroidal anti-inflammatory drug use.

For breast cancer analyses: models were further adjusted for menopausal hormone therapy use, oral contraceptive use, parity and age at first birth, age at menarche, BMI and menopause status (except when menopause status was subgroup of interest).

For prostate cancer analyses: models were further adjusted for marital status.

χ^2^ represents improvement of model fit and p-value represents heterogeneity between subgroups obtained from likelihood ratio tests for including an interaction term between subgroup of interest and calcium intake (modelled as a 300 mg/day increase) into the model.

Abbreviations: BMI, body mass index; CI, confidence interval; g/day, grams per day; HR, hazard ratio; kg/m^2^, kilograms per metre squared; N, number of participants.
